# Supplementary figures and images for: BBSome-deficient cells activate intraciliary CDC42 to trigger actin-dependent ciliary ectocytosis
Source: EMBO Rep. 2024 Nov 25;26(1):36–60. doi: 10.1038/s44319-024-00326-z (PMC11724091; doi:10.1038/s44319-024-00326-z)

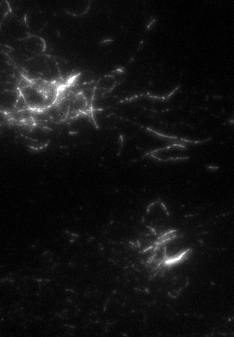

Supplement: Supplementary file 6 — Source data Fig. 1 [file 44319_2024_326_MOESM6_ESM.zip › Source data F1/A/MAX_RPE1 BBS4KO_11_R3D.tif]

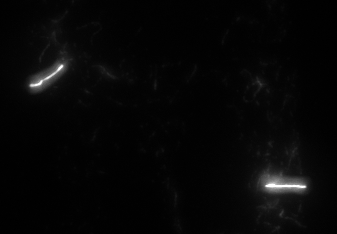

Supplement: Supplementary file 6 — Source data Fig. 1 [file 44319_2024_326_MOESM6_ESM.zip › Source data F1/A/MAX_RPE1 wt_08_R3D.tif]

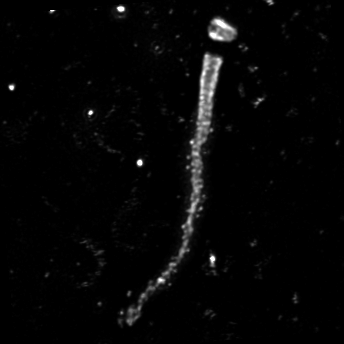

Supplement: Supplementary file 6 — Source data Fig. 1 [file 44319_2024_326_MOESM6_ESM.zip › Source data F1/C/ExM_RPE1 BBS4ko 010_decon_AcTub-scalebar.tif]

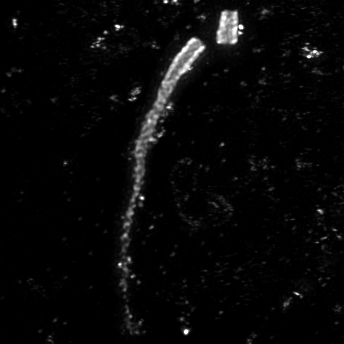

Supplement: Supplementary file 6 — Source data Fig. 1 [file 44319_2024_326_MOESM6_ESM.zip › Source data F1/C/ExM_RPE1 BBS4ko 011_decon_AcTub-scalebar.tif]

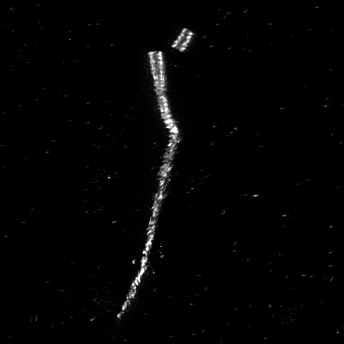

Supplement: Supplementary file 6 — Source data Fig. 1 [file 44319_2024_326_MOESM6_ESM.zip › Source data F1/C/ExM_RPE1 WT 06_decon_AcTub-scalebar.tif]

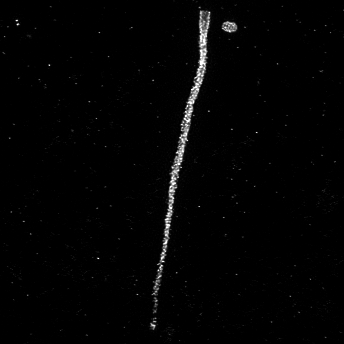

Supplement: Supplementary file 6 — Source data Fig. 1 [file 44319_2024_326_MOESM6_ESM.zip › Source data F1/C/ExM_RPE1 WT 07_decon_AcTub-scalebar.tif]

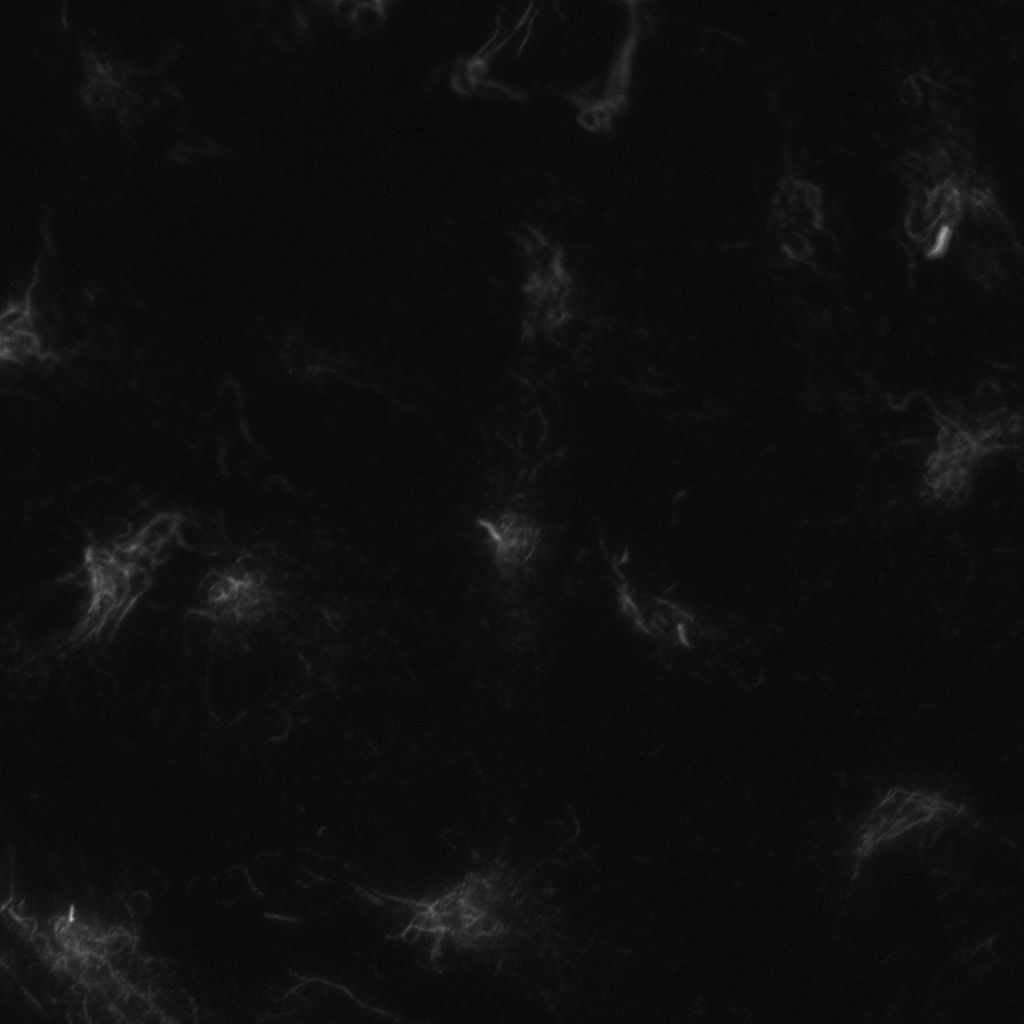

Supplement: Supplementary file 6 — Source data Fig. 1 [file 44319_2024_326_MOESM6_ESM.zip › Source data F1/D/RPE1_BBS1ko_AcTubAF488_PhallTexasRed_control.tif]

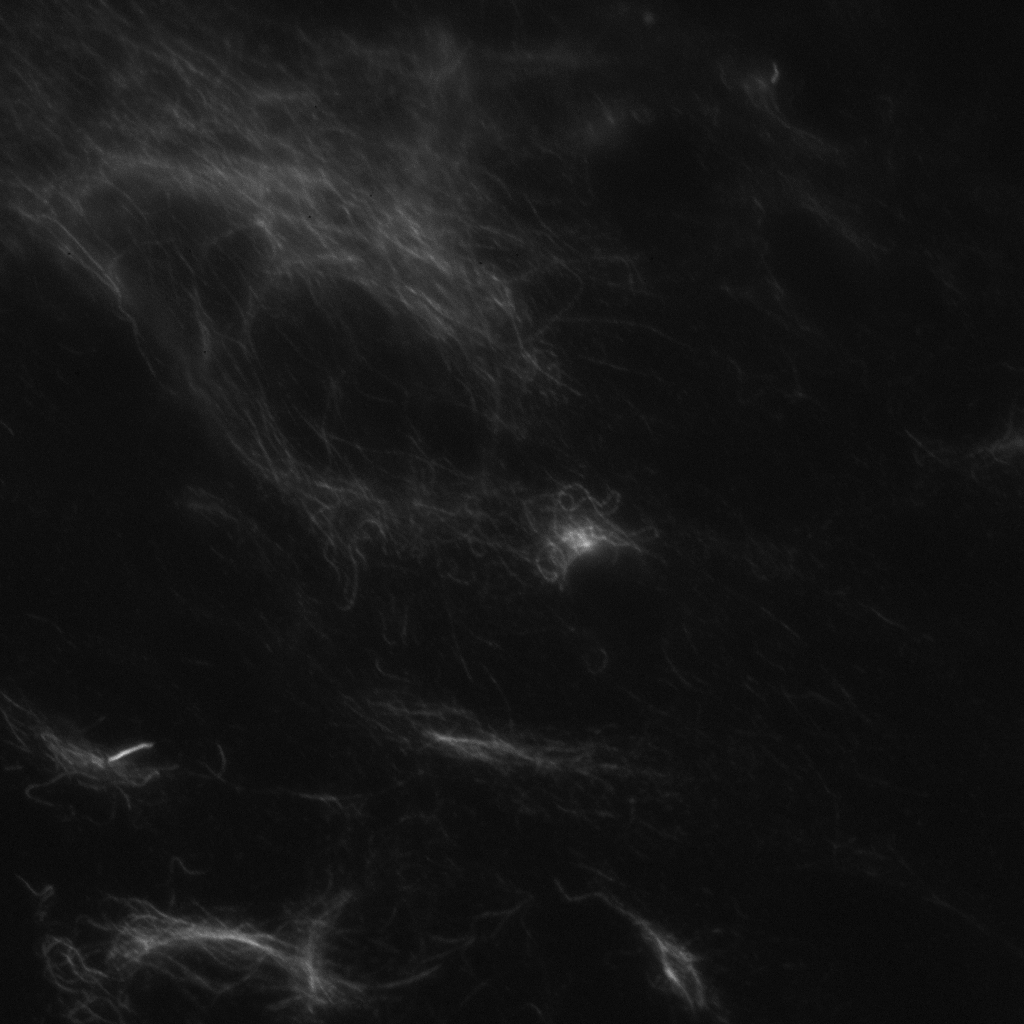

Supplement: Supplementary file 6 — Source data Fig. 1 [file 44319_2024_326_MOESM6_ESM.zip › Source data F1/D/RPE1_BBS1ko_AcTubAF488_PhallTexasRed_ML141 2h.tif]

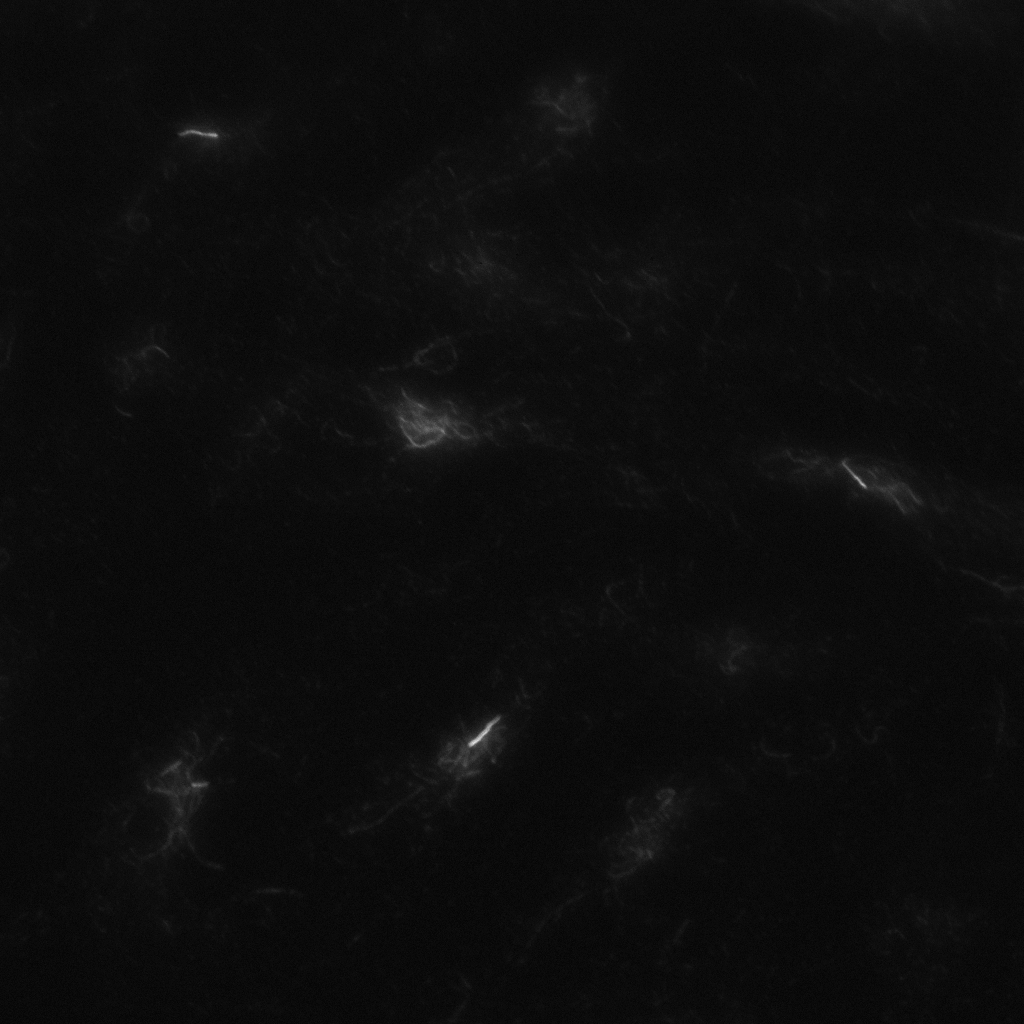

Supplement: Supplementary file 6 — Source data Fig. 1 [file 44319_2024_326_MOESM6_ESM.zip › Source data F1/D/RPE1_BBS1ko_AcTubAF488_PhallTexasRed_Y27632 2h.tif]

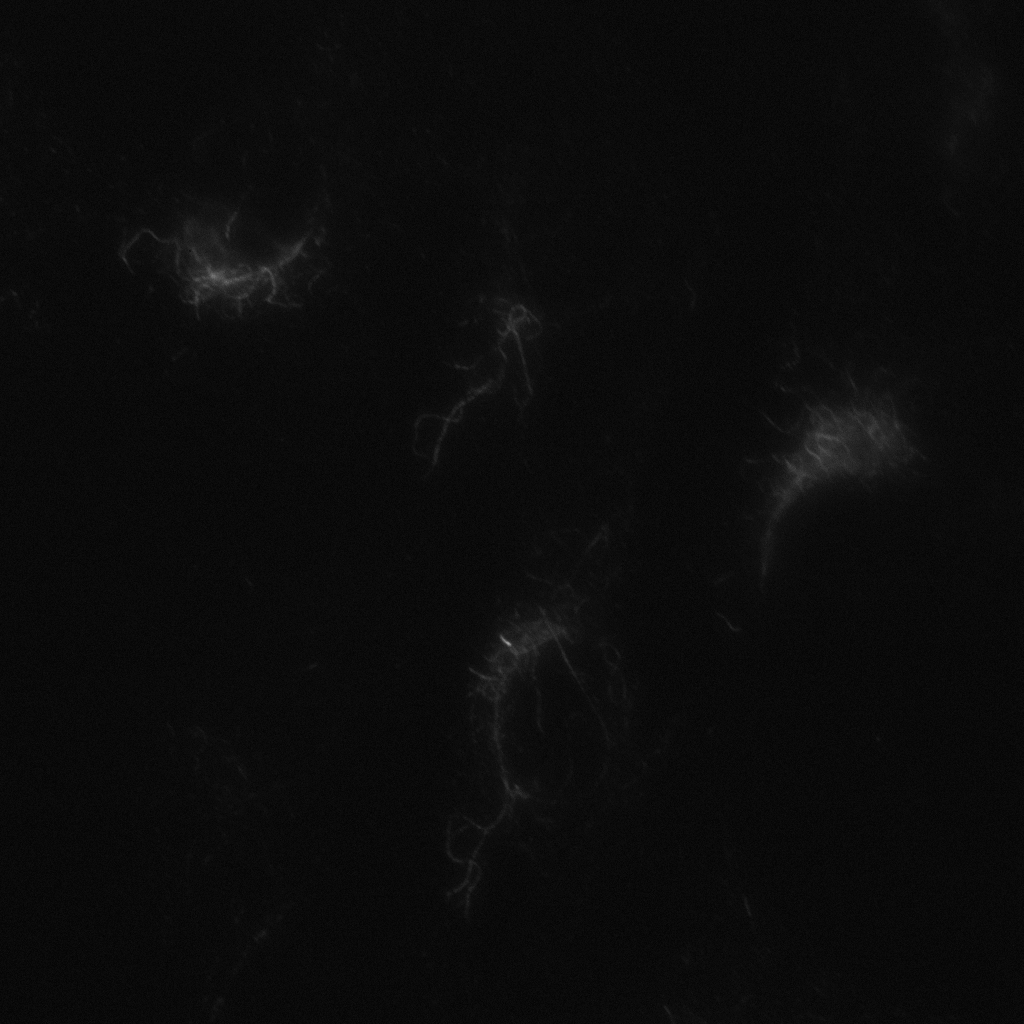

Supplement: Supplementary file 6 — Source data Fig. 1 [file 44319_2024_326_MOESM6_ESM.zip › Source data F1/D/RPE1_BBS4ko_AcTubAF488_PhallTexasRed_control.tif]

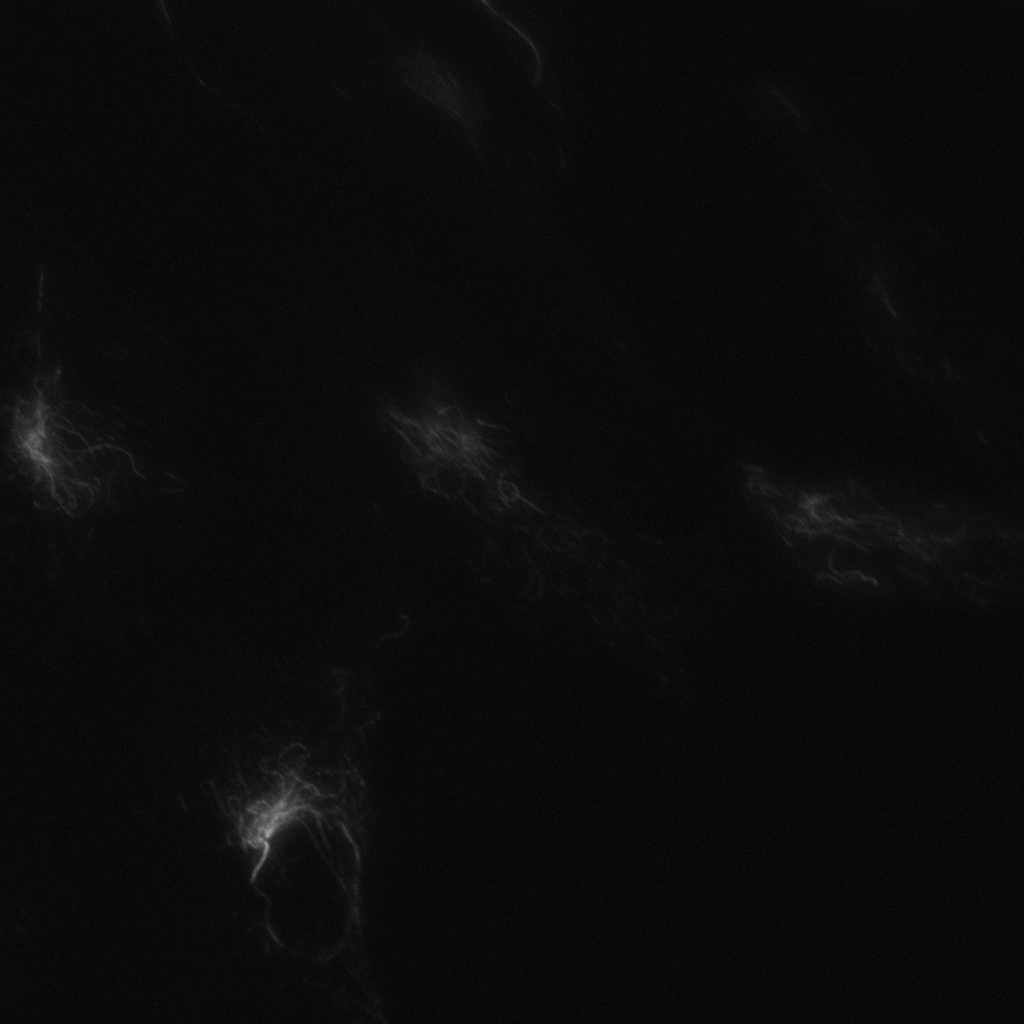

Supplement: Supplementary file 6 — Source data Fig. 1 [file 44319_2024_326_MOESM6_ESM.zip › Source data F1/D/RPE1_BBS4ko_AcTubAF488_PhallTexasRed_ML141 2h.tif]

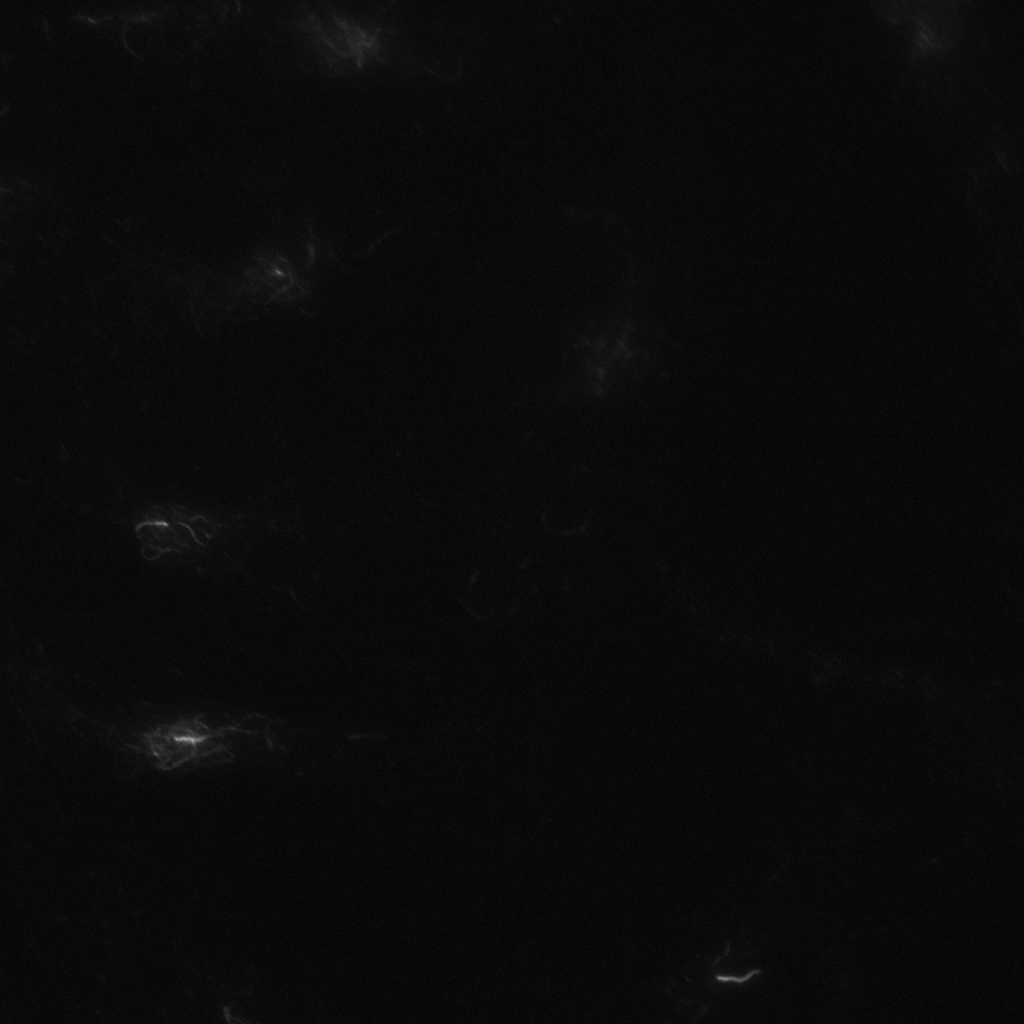

Supplement: Supplementary file 6 — Source data Fig. 1 [file 44319_2024_326_MOESM6_ESM.zip › Source data F1/D/RPE1_BBS4ko_AcTubAF488_PhallTexasRed_Y27632 2h.tif]

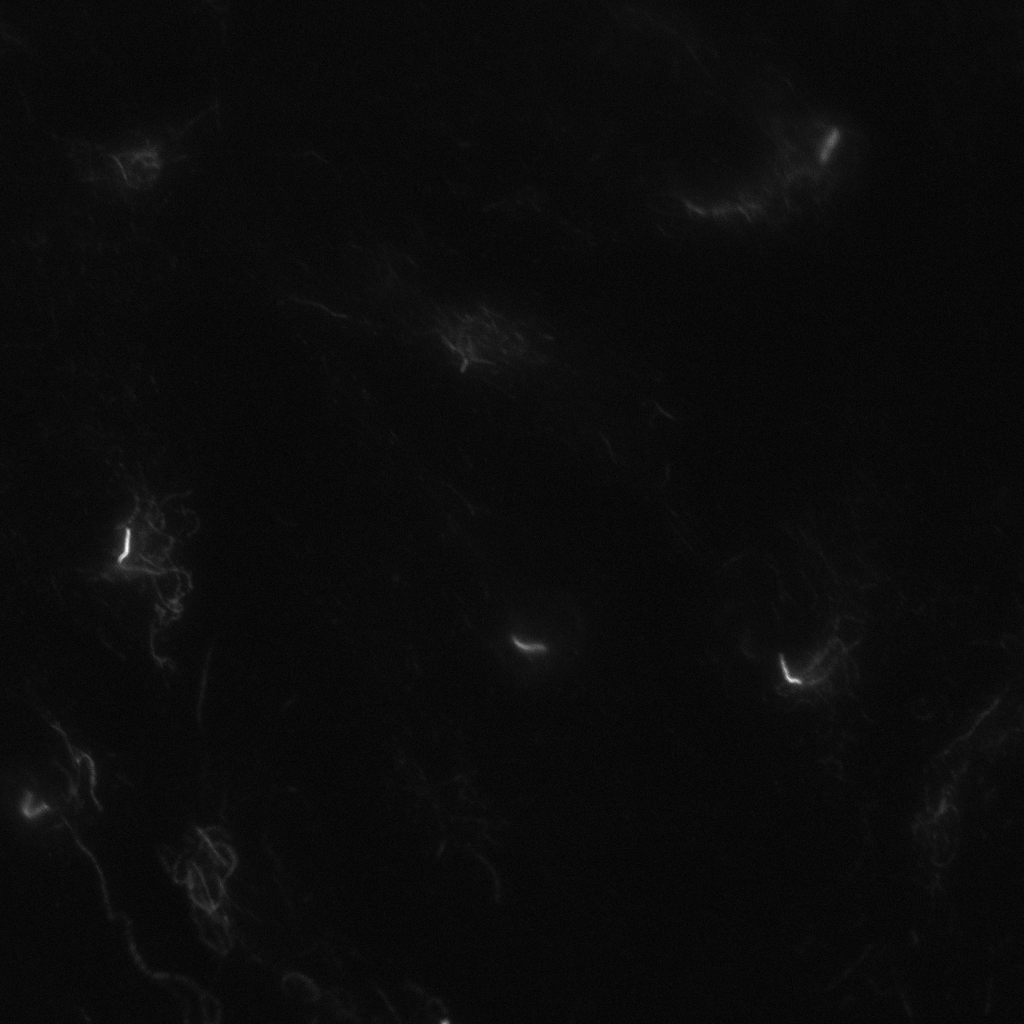

Supplement: Supplementary file 6 — Source data Fig. 1 [file 44319_2024_326_MOESM6_ESM.zip › Source data F1/D/RPE1_WT_AcTubAF488_PhallTexasRed_control.tif]

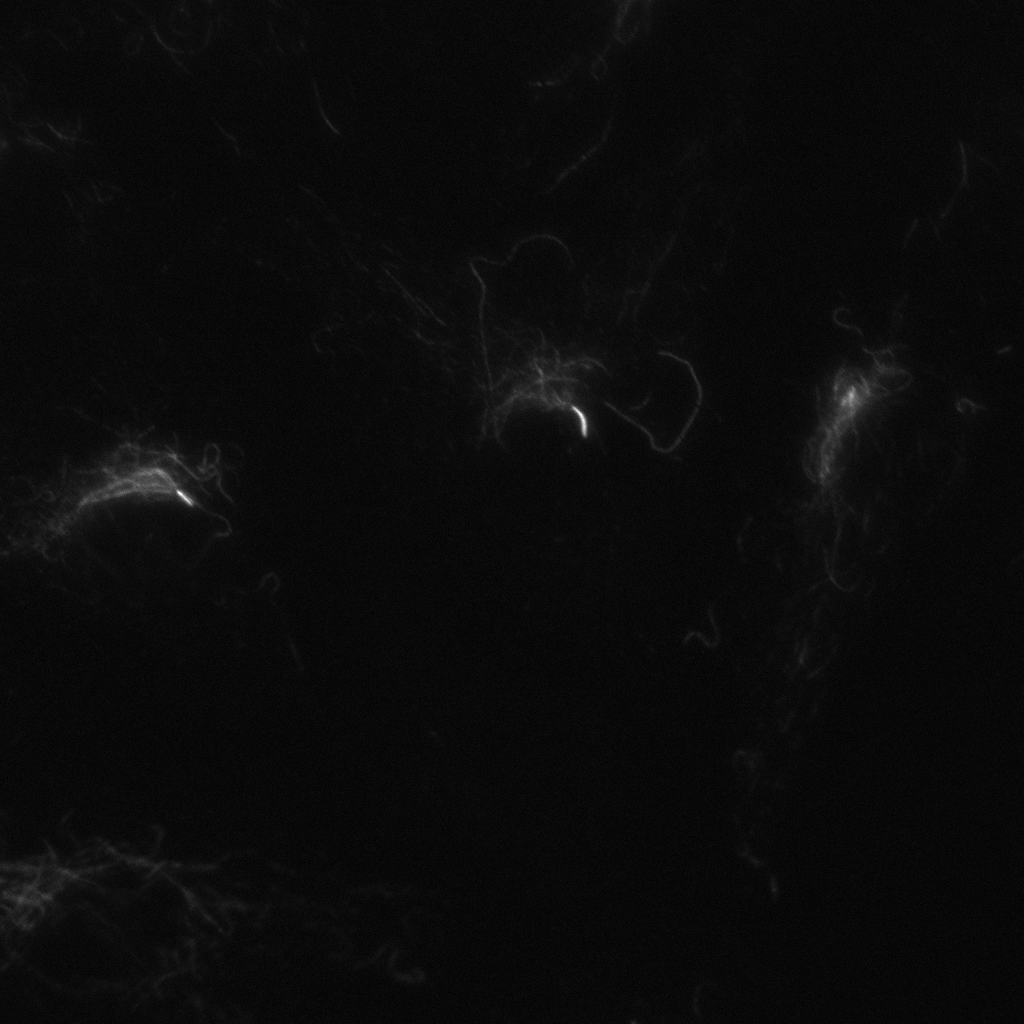

Supplement: Supplementary file 6 — Source data Fig. 1 [file 44319_2024_326_MOESM6_ESM.zip › Source data F1/D/RPE1_WT_AcTubAF488_PhallTexasRed_ML141 2h.tif]

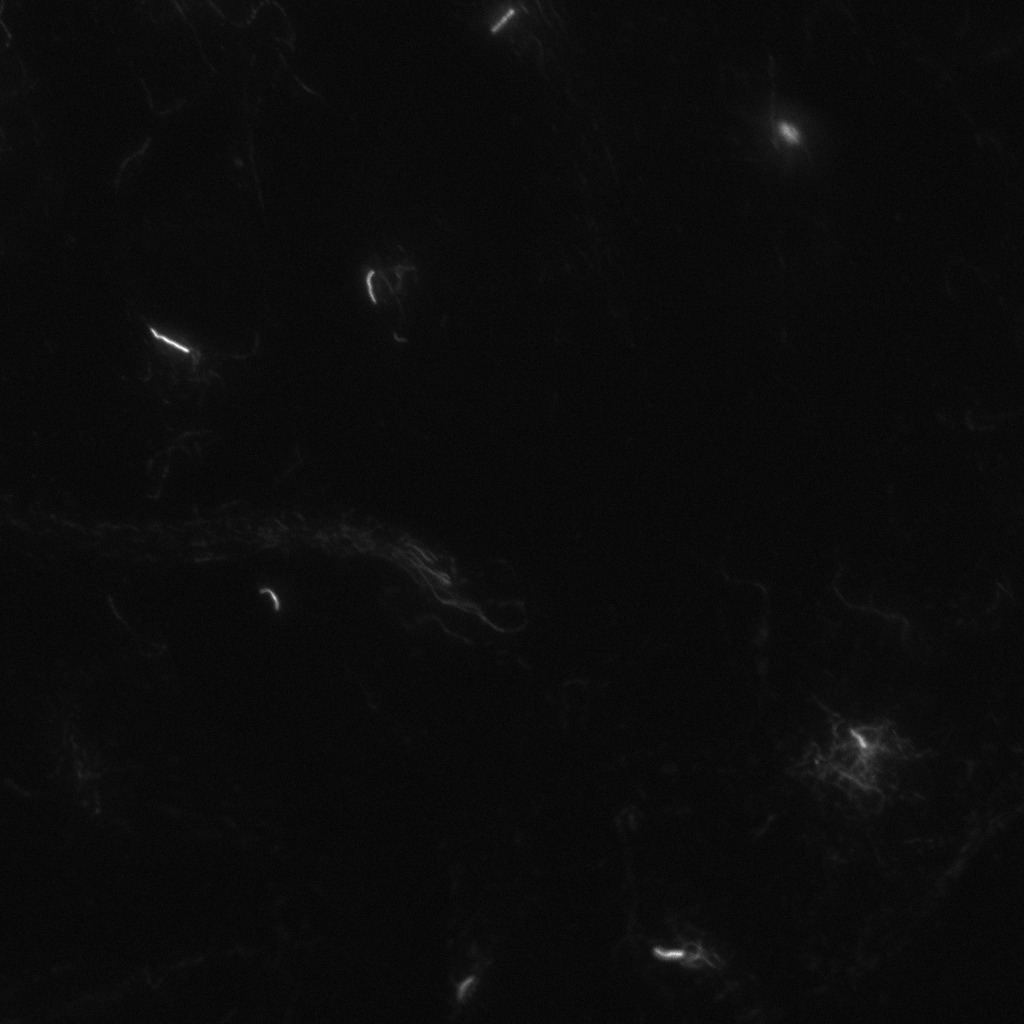

Supplement: Supplementary file 6 — Source data Fig. 1 [file 44319_2024_326_MOESM6_ESM.zip › Source data F1/D/RPE1_WT_AcTubAF488_PhallTexasRed_Y27632 2h.tif]

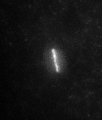

Supplement: Supplementary file 6 — Source data Fig. 1 [file 44319_2024_326_MOESM6_ESM.zip › Source data F1/I/MEF Bbs4KO_ARL13B488actub647PhallTexRed_DMSO 2h.tif]

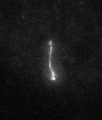

Supplement: Supplementary file 6 — Source data Fig. 1 [file 44319_2024_326_MOESM6_ESM.zip › Source data F1/I/MEF Bbs4KO_ARL13B488actub647PhallTexRed_ML141 2h.tif]

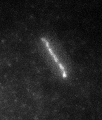

Supplement: Supplementary file 6 — Source data Fig. 1 [file 44319_2024_326_MOESM6_ESM.zip › Source data F1/I/MEF WT_ARL13B488actub647PhallTexRed_DMSO 2h.tif]

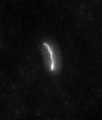

Supplement: Supplementary file 6 — Source data Fig. 1 [file 44319_2024_326_MOESM6_ESM.zip › Source data F1/I/MEF WT_ARL13B488actub647PhallTexRed_ML141 2h.tif]

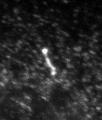

Supplement: Supplementary file 7 — Source data Fig. 2 [file 44319_2024_326_MOESM7_ESM.zip › Source data F2/A/MAX_MEF_Bbs4 KO_GPR161af488_Acetubaf555_nt.tif]

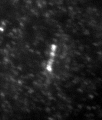

Supplement: Supplementary file 7 — Source data Fig. 2 [file 44319_2024_326_MOESM7_ESM.zip › Source data F2/A/MAX_MEF_Bbs4 KO_GPR161af488_Acetubaf555_SAG2h 1.tif]

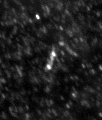

Supplement: Supplementary file 7 — Source data Fig. 2 [file 44319_2024_326_MOESM7_ESM.zip › Source data F2/A/MAX_MEF_Bbs4 KO_GPR161af488_Acetubaf555_SAG2h 2.tif]

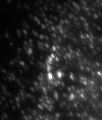

Supplement: Supplementary file 7 — Source data Fig. 2 [file 44319_2024_326_MOESM7_ESM.zip › Source data F2/A/MAX_MEF_WT_GPR161af488_Acetubaf555_nt.tif]

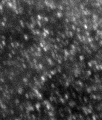

Supplement: Supplementary file 7 — Source data Fig. 2 [file 44319_2024_326_MOESM7_ESM.zip › Source data F2/A/MAX_MEF_WT_GPR161af488_Acetubaf555_SAG 2h.tif]

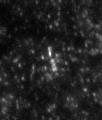

Supplement: Supplementary file 7 — Source data Fig. 2 [file 44319_2024_326_MOESM7_ESM.zip › Source data F2/D/MAX_MEF_Bbs4 KO_GPR161af488_Acetubaf555_ML141_nt.tif]

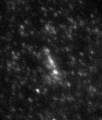

Supplement: Supplementary file 7 — Source data Fig. 2 [file 44319_2024_326_MOESM7_ESM.zip › Source data F2/D/MAX_MEF_Bbs4 KO_GPR161af488_Acetubaf555_ML141_SAG 2h.tif]

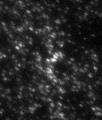

Supplement: Supplementary file 7 — Source data Fig. 2 [file 44319_2024_326_MOESM7_ESM.zip › Source data F2/D/MAX_MEF_WT_GPR161af488_Acetubaf555_ML141_nt.tif]

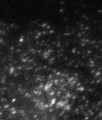

Supplement: Supplementary file 7 — Source data Fig. 2 [file 44319_2024_326_MOESM7_ESM.zip › Source data F2/D/MAX_MEF_WT_GPR161af488_Acetubaf555_ML141_SAG 2h.tif]

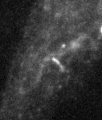

Supplement: Supplementary file 7 — Source data Fig. 2 [file 44319_2024_326_MOESM7_ESM.zip › Source data F2/F/MAX_MEF_Bbs4 KO_N-GFPcdc42DN_GPR161af555actub647_nt.tif]

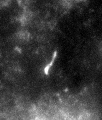

Supplement: Supplementary file 7 — Source data Fig. 2 [file 44319_2024_326_MOESM7_ESM.zip › Source data F2/F/MAX_MEF_Bbs4 KO_N-GFPcdc42DN_GPR161af555actub647_SAG 2h.tif]

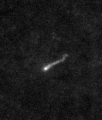

Supplement: Supplementary file 7 — Source data Fig. 2 [file 44319_2024_326_MOESM7_ESM.zip › Source data F2/F/MAX_MEF_Bbs4 KO_N-GFPcdc42WT_GPR161af555actub647_nt.tif]

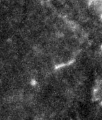

Supplement: Supplementary file 7 — Source data Fig. 2 [file 44319_2024_326_MOESM7_ESM.zip › Source data F2/F/MAX_MEF_Bbs4 KO_N-GFPcdc42WT_GPR161af555actub647_SAG 2h.tif]

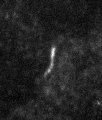

Supplement: Supplementary file 7 — Source data Fig. 2 [file 44319_2024_326_MOESM7_ESM.zip › Source data F2/F/MAX_MEF_WT_N-GFPcdc42DN_GPR161af555actub647_nt.tif]

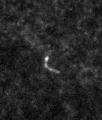

Supplement: Supplementary file 7 — Source data Fig. 2 [file 44319_2024_326_MOESM7_ESM.zip › Source data F2/F/MAX_MEF_WT_N-GFPcdc42DN_GPR161af555actub647_SAG 2h.tif]

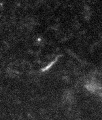

Supplement: Supplementary file 7 — Source data Fig. 2 [file 44319_2024_326_MOESM7_ESM.zip › Source data F2/F/MAX_MEF_WT_N-GFPcdc42WT_GPR161af555actub647_nt.tif]

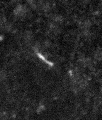

Supplement: Supplementary file 7 — Source data Fig. 2 [file 44319_2024_326_MOESM7_ESM.zip › Source data F2/F/MAX_MEF_WT_N-GFPcdc42WT_GPR161af555actub647_SAG 2h.tif]

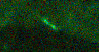

Supplement: Supplementary file 8 — Source data Fig. 3 [file 44319_2024_326_MOESM8_ESM.zip › Source data F3/C/N-Raichu-CDC42 MEF Bbs4KO nt/raichu 4ko nt 1_range 1 to 3.5ns-1.tif]

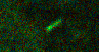

Supplement: Supplementary file 8 — Source data Fig. 3 [file 44319_2024_326_MOESM8_ESM.zip › Source data F3/C/N-Raichu-CDC42 MEF Bbs4KO nt/raichu 4KO nt 2_range 1 to 3.5ns-1.tif]

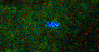

Supplement: Supplementary file 8 — Source data Fig. 3 [file 44319_2024_326_MOESM8_ESM.zip › Source data F3/C/N-Raichu-CDC42 MEF Bbs4KO nt/raichu 4KO nt 3_range 1 to 3.5ns-1.tif]

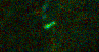

Supplement: Supplementary file 8 — Source data Fig. 3 [file 44319_2024_326_MOESM8_ESM.zip › Source data F3/C/N-Raichu-CDC42 MEF Bbs4KO SAG 2h/raichu 4ko sag 4_range 1 to 3.5ns-1.tif]

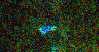

Supplement: Supplementary file 8 — Source data Fig. 3 [file 44319_2024_326_MOESM8_ESM.zip › Source data F3/C/N-Raichu-CDC42 MEF Bbs4KO SAG 2h/raichu 4KO sag 5_range 1 to 3.5ns-1.tif]

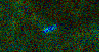

Supplement: Supplementary file 8 — Source data Fig. 3 [file 44319_2024_326_MOESM8_ESM.zip › Source data F3/C/N-Raichu-CDC42 MEF Bbs4KO SAG 2h/raichu 4KO sag 7_range 1 to 3.5ns-1.tif]

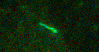

Supplement: Supplementary file 8 — Source data Fig. 3 [file 44319_2024_326_MOESM8_ESM.zip › Source data F3/C/N-Raichu-CDC42 MEFwt nt/raichu wt nt 2_range 1 to 3.5ns-1.tif]

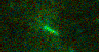

Supplement: Supplementary file 8 — Source data Fig. 3 [file 44319_2024_326_MOESM8_ESM.zip › Source data F3/C/N-Raichu-CDC42 MEFwt nt/raichu wt nt 3_range 1 to 3.5ns-1.tif]

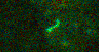

Supplement: Supplementary file 8 — Source data Fig. 3 [file 44319_2024_326_MOESM8_ESM.zip › Source data F3/C/N-Raichu-CDC42 MEFwt nt/raichu wt nt 7_range 1 to 3.5ns-1.tif]

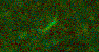

Supplement: Supplementary file 8 — Source data Fig. 3 [file 44319_2024_326_MOESM8_ESM.zip › Source data F3/C/N-Raichu-CDC42 MEFwt SAG 2h/raichu wt sag 2_range 1 to 3.5ns-1.tif]

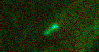

Supplement: Supplementary file 8 — Source data Fig. 3 [file 44319_2024_326_MOESM8_ESM.zip › Source data F3/C/N-Raichu-CDC42 MEFwt SAG 2h/raichu wt sag 5_range 1 to 3.5ns-1.tif]

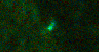

Supplement: Supplementary file 8 — Source data Fig. 3 [file 44319_2024_326_MOESM8_ESM.zip › Source data F3/C/N-Raichu-CDC42 MEFwt SAG 2h/raichu wt sag 6_range 1 to 3.5ns-1.tif]

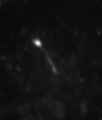

Supplement: Supplementary file 9 — Source data Fig. 4 [file 44319_2024_326_MOESM9_ESM.zip › Source data F4/A/MAX_MEF Bbs4 KO_SMO488_GPR161mCherry_ActubCy5_nt_ML141.tif]

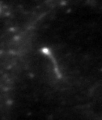

Supplement: Supplementary file 9 — Source data Fig. 4 [file 44319_2024_326_MOESM9_ESM.zip › Source data F4/A/MAX_MEF Bbs4 KO_SMO488_GPR161mCherry_ActubCy5_SAG_DMSO 2.tif]

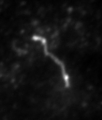

Supplement: Supplementary file 9 — Source data Fig. 4 [file 44319_2024_326_MOESM9_ESM.zip › Source data F4/A/MAX_MEF Bbs4 KO_SMO488_GPR161mCherry_ActubCy5_SAG_ML141 1.tif]

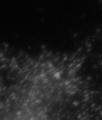

Supplement: Supplementary file 9 — Source data Fig. 4 [file 44319_2024_326_MOESM9_ESM.zip › Source data F4/A/MAX_MEF WT_SMO488_GPR161mCherry_ActubCy5_nt_DMSO.tif]

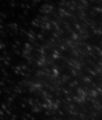

Supplement: Supplementary file 9 — Source data Fig. 4 [file 44319_2024_326_MOESM9_ESM.zip › Source data F4/A/MAX_MEF WT_SMO488_GPR161mCherry_ActubCy5_nt_ML141.tif]

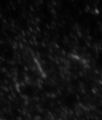

Supplement: Supplementary file 9 — Source data Fig. 4 [file 44319_2024_326_MOESM9_ESM.zip › Source data F4/A/MAX_MEF WT_SMO488_GPR161mCherry_ActubCy5_SAG_DMSO 1.tif]

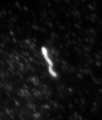

Supplement: Supplementary file 9 — Source data Fig. 4 [file 44319_2024_326_MOESM9_ESM.zip › Source data F4/A/MAX_MEF WT_SMO488_GPR161mCherry_ActubCy5_SAG_DMSO 2.tif]

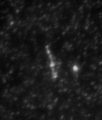

Supplement: Supplementary file 9 — Source data Fig. 4 [file 44319_2024_326_MOESM9_ESM.zip › Source data F4/A/MAX_MEF WT_SMO488_GPR161mCherry_ActubCy5_SAG_ML141 1.tif]

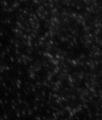

Supplement: Supplementary file 9 — Source data Fig. 4 [file 44319_2024_326_MOESM9_ESM.zip › Source data F4/A/MAX_MEF WT_SMO488_GPR161mCherry_ActubCy5_SAG_ML141 2.tif]

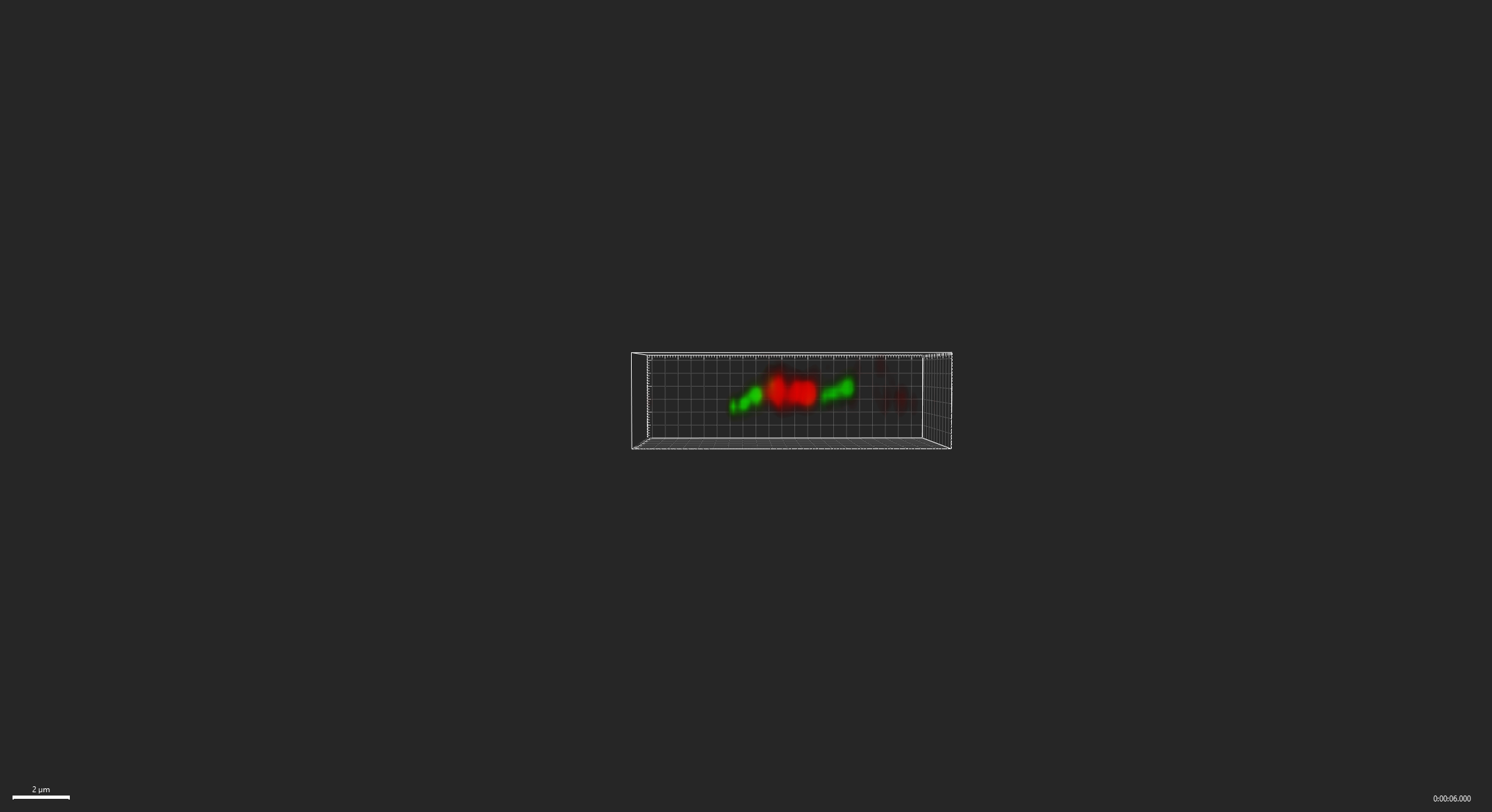

Supplement: Supplementary file 10 — Source data Fig. 5 [file 44319_2024_326_MOESM10_ESM.zip › Source data F5/B/MEF Bbs4ko ARL13BmNG_LifeActRFP_DMSO_SAG Rotated.tif]

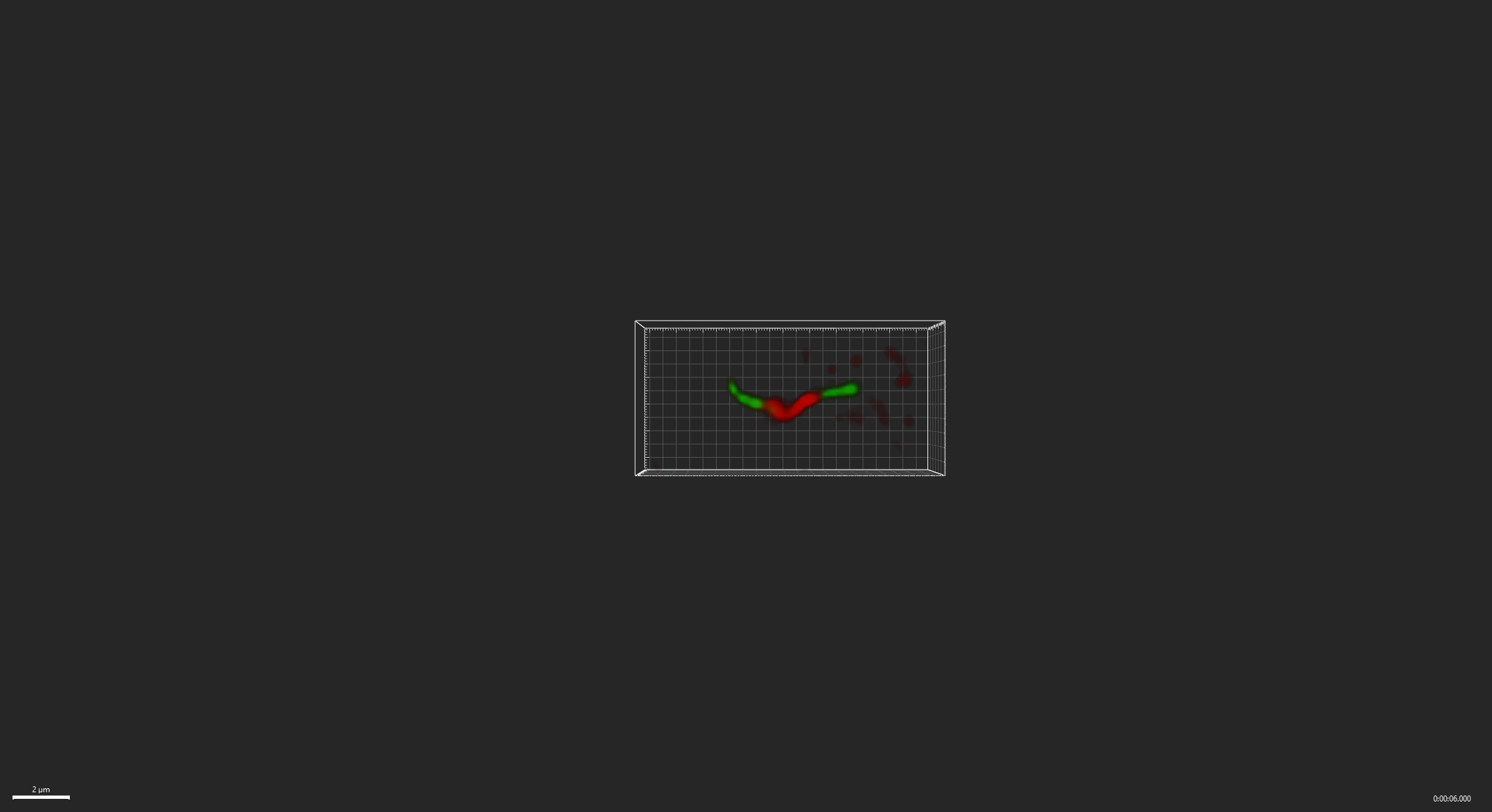

Supplement: Supplementary file 10 — Source data Fig. 5 [file 44319_2024_326_MOESM10_ESM.zip › Source data F5/B/MEF Bbs4ko ARL13BmNG_LifeActRFP_DMSO_SAG.tif]

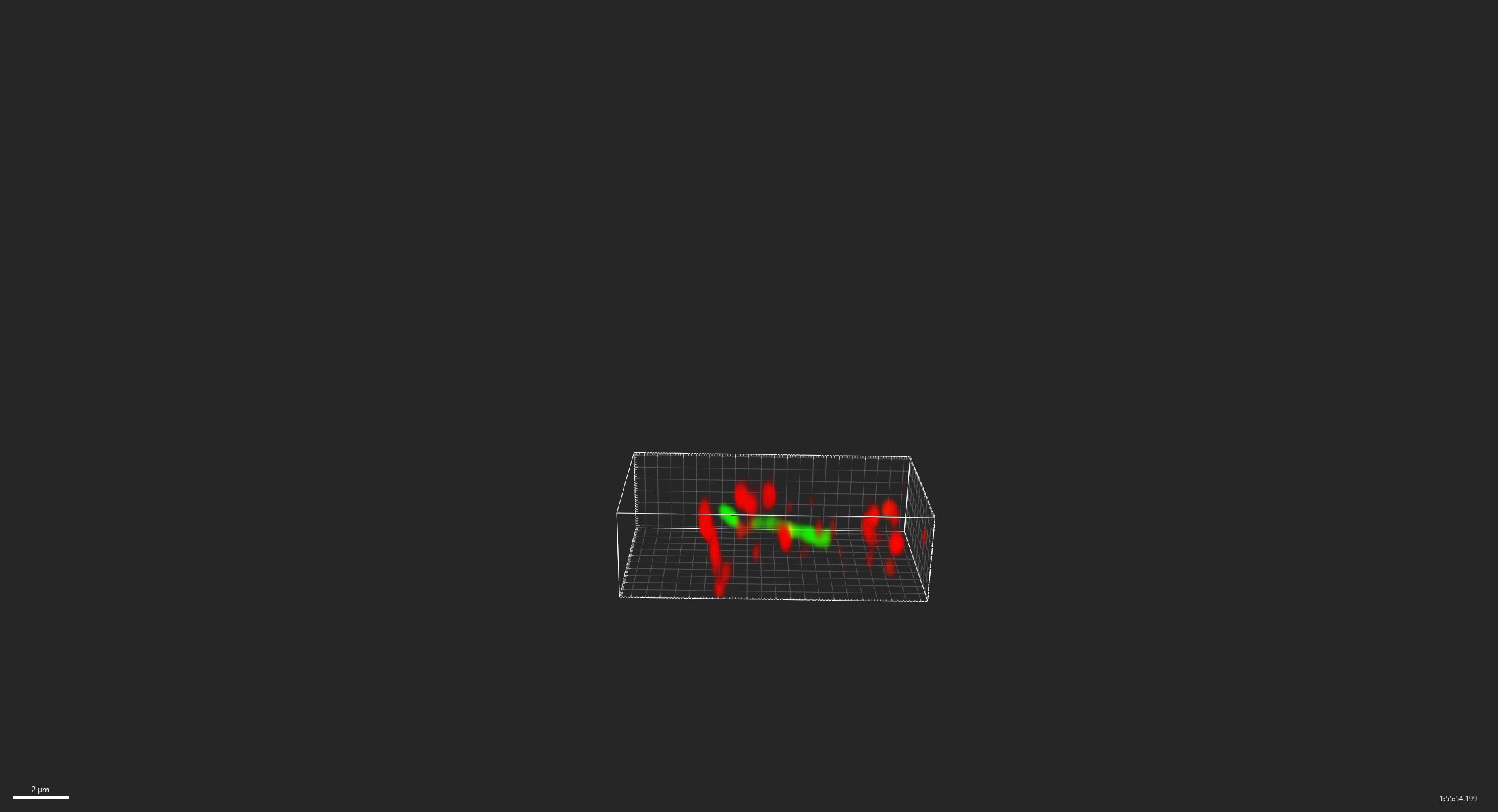

Supplement: Supplementary file 10 — Source data Fig. 5 [file 44319_2024_326_MOESM10_ESM.zip › Source data F5/B/MEF Bbs4ko ARL13BmNG_LifeActRFP_ML141_SAG rotated.tif]

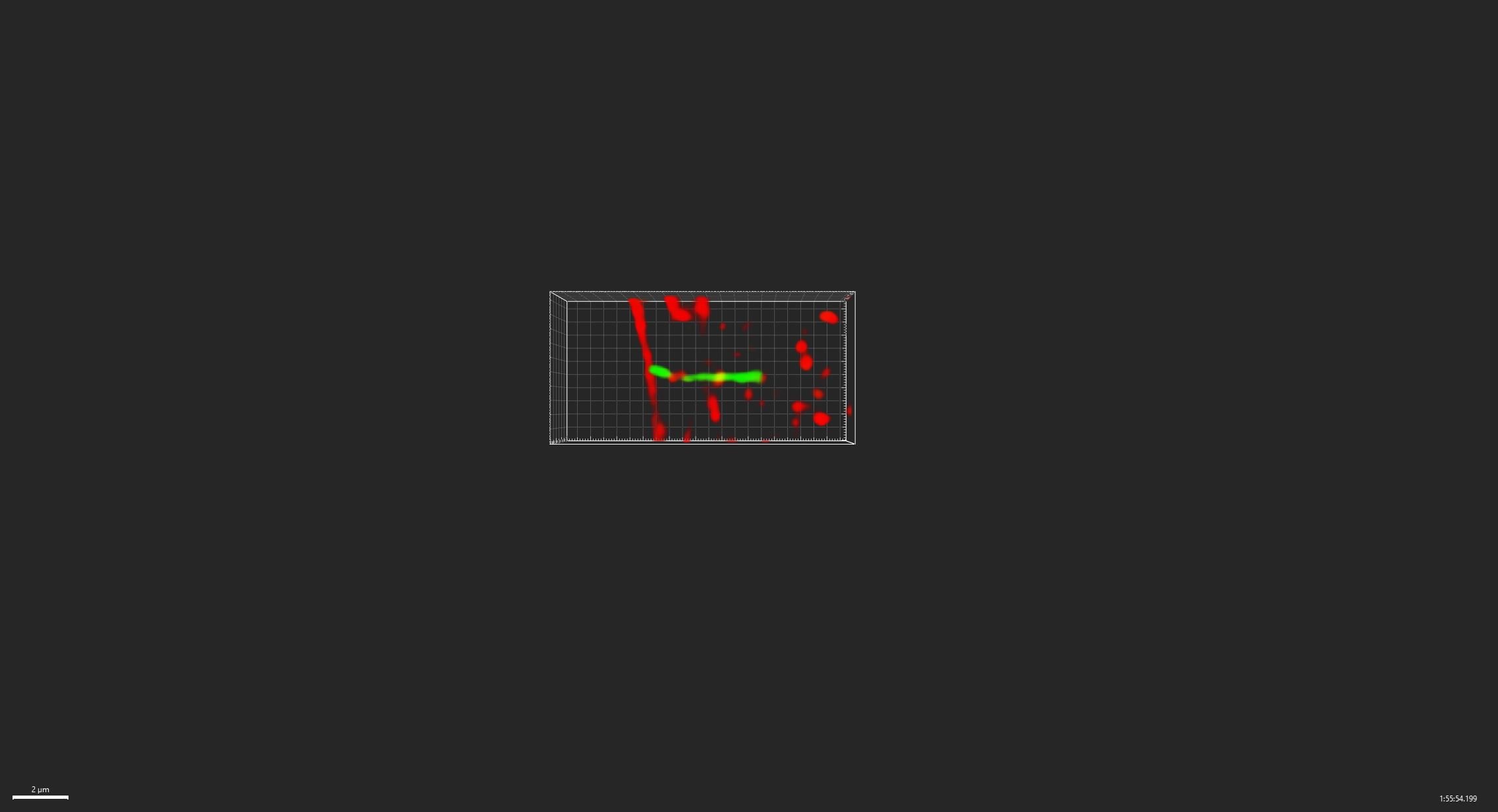

Supplement: Supplementary file 10 — Source data Fig. 5 [file 44319_2024_326_MOESM10_ESM.zip › Source data F5/B/MEF Bbs4ko ARL13BmNG_LifeActRFP_ML141_SAG.tif]

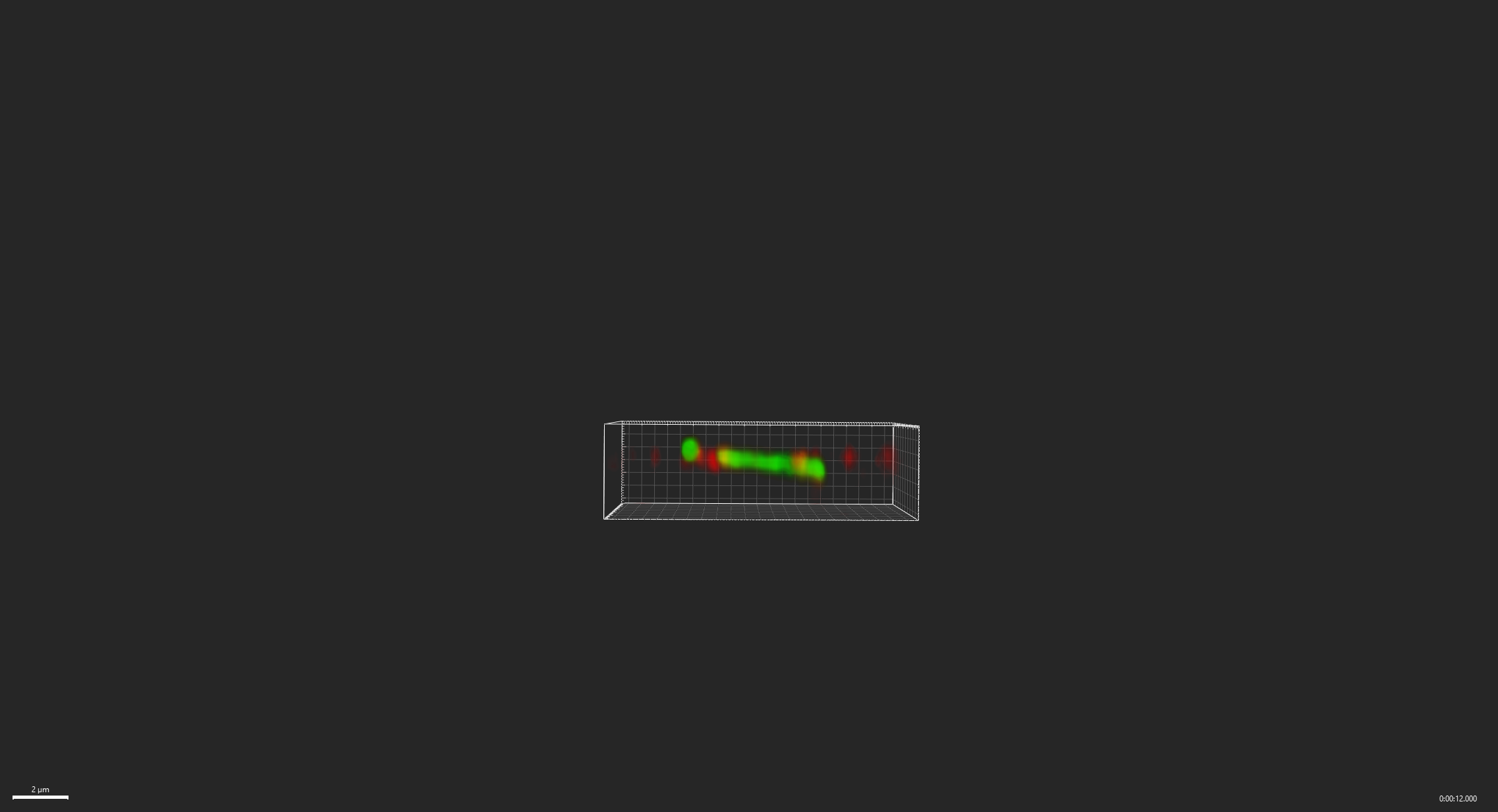

Supplement: Supplementary file 10 — Source data Fig. 5 [file 44319_2024_326_MOESM10_ESM.zip › Source data F5/B/MEF WT ARL13BmNG_LifeActRFP_DMSO_SAG Rotated.tif]

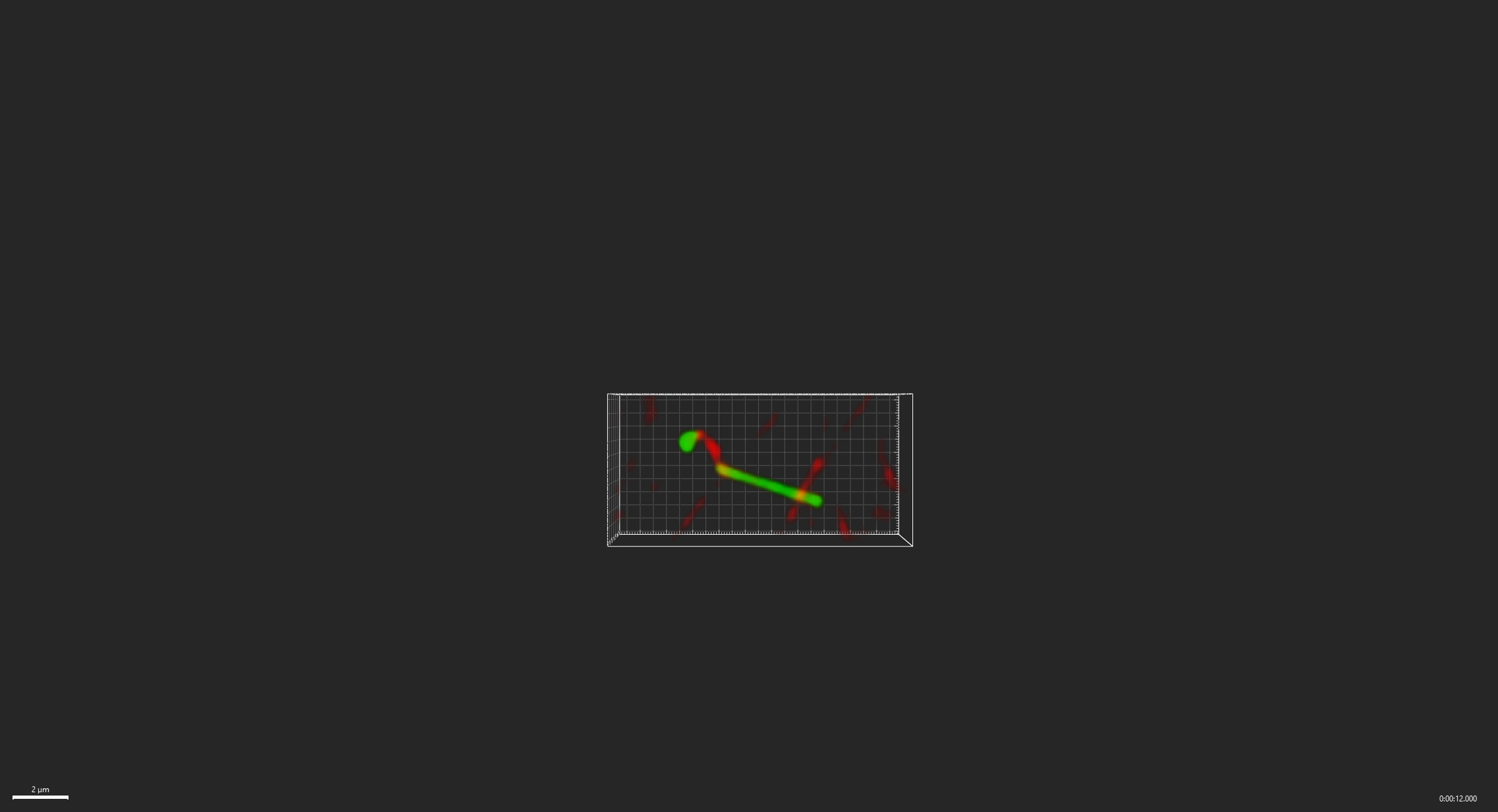

Supplement: Supplementary file 10 — Source data Fig. 5 [file 44319_2024_326_MOESM10_ESM.zip › Source data F5/B/MEF WT ARL13BmNG_LifeActRFP_DMSO_SAG.tif]

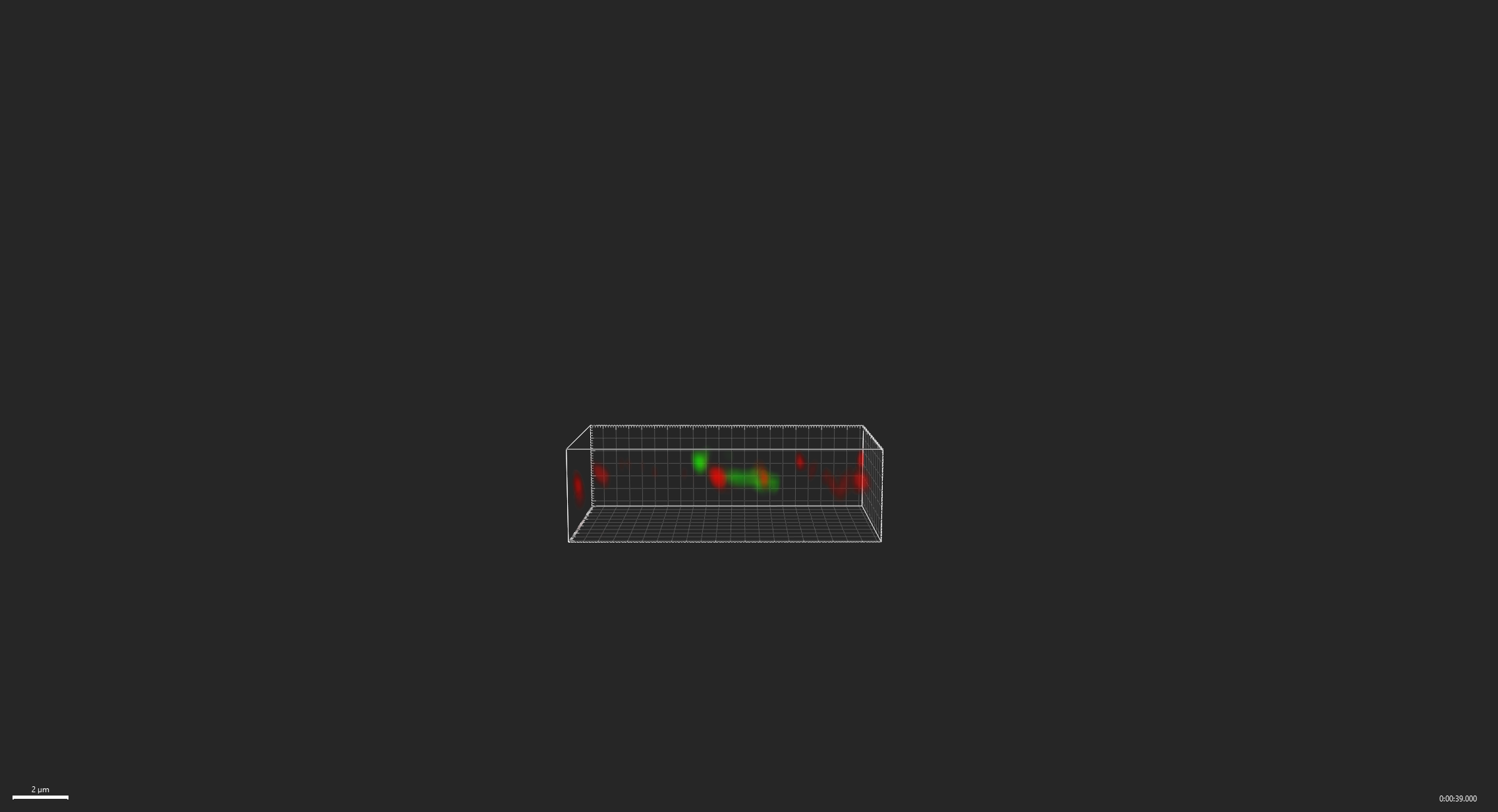

Supplement: Supplementary file 10 — Source data Fig. 5 [file 44319_2024_326_MOESM10_ESM.zip › Source data F5/B/MEF WT ARL13BmNG_LifeActRFP_ML141_SAG Rotated.tif]

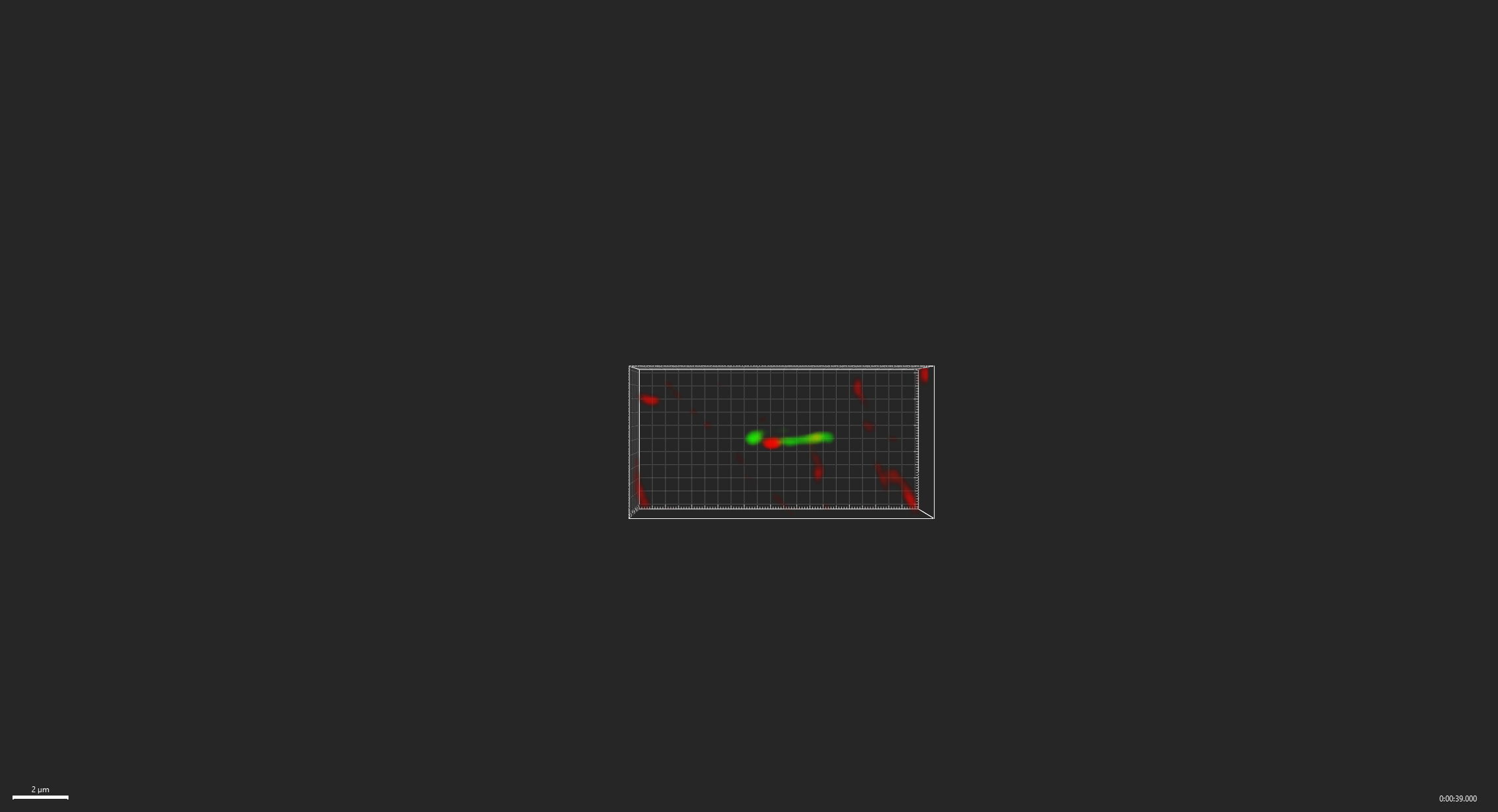

Supplement: Supplementary file 10 — Source data Fig. 5 [file 44319_2024_326_MOESM10_ESM.zip › Source data F5/B/MEF WT ARL13BmNG_LifeActRFP_ML141_SAG.tif]
