# Supplementary material for: BBSome-deficient cells activate intraciliary CDC42 to trigger actin-dependent ciliary ectocytosis
Source: EMBO Rep. 2024 Nov 25;26(1):36–60. doi: 10.1038/s44319-024-00326-z (PMC11724091; doi:10.1038/s44319-024-00326-z)
Supplement: Supplementary file 11 — Expanded View Figures [file 44319_2024_326_MOESM11_ESM.pdf]

Expanded View Figures

Figure EV1. Actin regulators regulate the cilia length in different manner.

(**A**) Expansion microscopy of the cilia axoneme of the *BBS1*$^{KO/KO}$ and *BBS7*$^{KO/KO}$ RPE1 cells visualized by staining with antibody to acetylated tubulin. White arrows point to bulges at the cilia tips. Scale bar, 2 µm. (**B**) The graph shows the frequency of bulges at the cilia tips visualized by expansion microscopy of cilia in WT and *BBS4*$^{KO/KO}$ RPE1 cells. The number of observed bulges out of the total count of imaged cilia is shown. Statistical analysis was done using the contingency table and one-sided Chi-square test. (**C**) Representative micrographs of the cilia visualized by staining with antibody to acetylated tubulin (Ac-tub) and staining of actin cytoskeleton with Phalloidin-TexasRed in the WT RPE1 cells treated with inhibitors of ROCK1/RHOA (Y27632), CDC42 (ML141), RAC1 (*CAS*1177865-17-6 (CAS)), ARP2/3 (CK666), and actin polymerization (Cytochalasin D) for 2 h. Scale bar, 5 µm. (**D**) Quantification of the cilia length based on acetylated tubulin signal in the WT and *BBS4*$^{KO/KO}$ RPE1 cells treated with DMSO or CK666 or Cytochalasin D (CytoD) for 2 h as in (**C**). Medians with interquartile range from three independent experiments ($n = 220$–320 cilia). (**E**) Quantification of the cilia length based on acetylated tubulin signal in the WT and *BBS4*$^{KO/KO}$ RPE1 cells treated with vehicle or CAS for 2 h as in (**C**). Medians with interquartile range from three independent experiments ($n = 250$–280 cilia). (**F**) Representative micrographs of the cilia visualized by staining with antibody to acetylated tubulin (Ac-tub) and ARL13B in the WT and *BBS4*$^{KO/KO}$ RPE1 cells treated with DMSO or ML141 for 2 h. Scale bar, 5 µm. (**G**) Quantification of the cilia length in WT, *BBS4*$^{KO/KO}$, and *BBS4*$^{KO/KO}$ RPE1 cells expressing YFP-BBS4 treated with DMSO or ML141 for 2 h and stained with antibodies to acetylated tubulin (Ac-tub) and ARL13B. Medians with interquartile range from three independent experiments ($n = 200$–250 cilia). (**H**) Quantification of the frequency of ARL13B foci at the cilia tips in WT, *BBS4*$^{KO/KO}$, and *BBS4*$^{KO/KO}$ RPE1 cells expressing YFP-BBS4 treated with DMSO or ML141 for 2 h in (**G**). Mean and SD of three independent experiments ($n = 200$–250 cilia). (**I**) Intensity plot profiles of the Ac-tub and ARL13B signal measured from the cilia base (B) to the cilia tip (T) in *Bbs4* KO MEFs treated with ML141 in Fig. 1I. The ARL13B signal extends (~0.5 µm) beyond the Ac-tub signal. (**J**) Representative chromatogram analysis of DNA sequencing data for WT ST2 and *Ift88* KO ST2 cell clones reveals a homozygous mutation characterized by the insertion of a T nucleotide between bases 248 and 249 of the *Ift88* gene (NM_009376.3, arrow). The position of the sgRNA and PAM sequence used for targeting is indicated. (**K**) Representative Western blot from total cell lysates prepared from WT MEFs, SAG/DMSO treated GPR161-mCherry WT MEFs (experiments #1–4 in Fig. 1K–M), WT and *Ift88* KO ST2 cells and *Ift88* KO ST2 cells expressing the IFT88-mNeonGreen and probed with antibodies to IFT88 and β-actin. The arrow highlights the IFT88 band (red star) corresponding to the expected size of 100 kDa, which is absent in the *Ift88* KO ST2 cells. Data information: Statistical significance was calculated using the one-sided Chi-square test (**B**), two-tailed Mann–Whitney test (**D**, **E**, **G**) and two-tailed paired t-test (**H**) and the obtained *p*-values are indicated. Merged micrographs show nuclei staining with DAPI—blue (**F**).

© The Author(s)

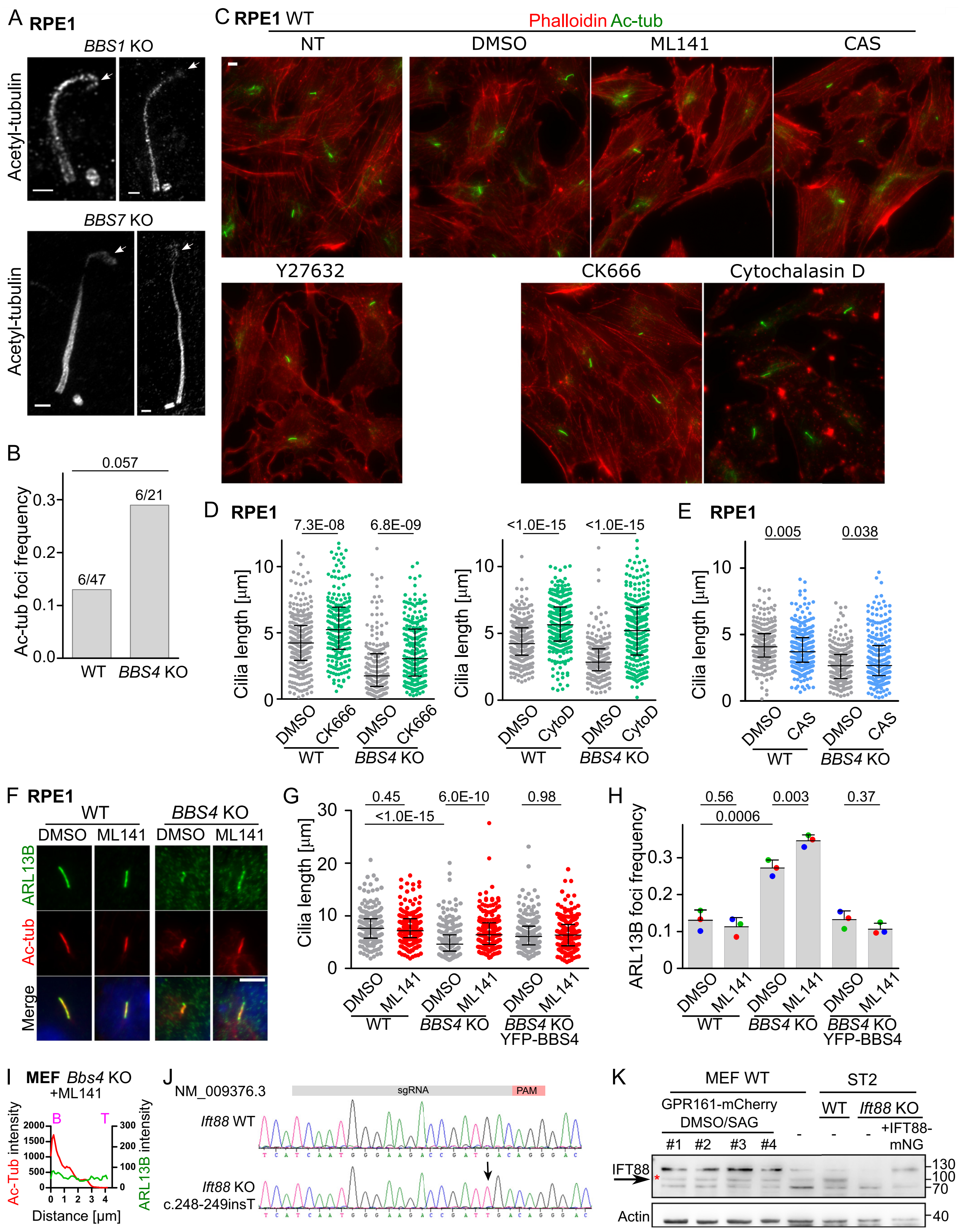
A RPE1
BBS1 KO
Acetyl-tubulin
BBS7 KO
Acetyl-tubulin
B
0.057
6/47
6/21
Ac-tub foci frequency
WT
BBS4 KO
C RPE1 WT
Phalloidin Ac-tub
NT
DMSO
ML141
CAS
Y27632
CK666
Cytochalasin D
D RPE1
7.3E-08
6.8E-09
<1.0E-15
<1.0E-15
Cilia length [µm]
DMSO
CK666
CytoD
WT
BBS4 KO
E RPE1
0.005
0.038
Cilia length [µm]
DMSO
CAS
WT
BBS4 KO
F RPE1
WT
BBS4 KO
DMSO ML141 DMSO ML141
ARL13B
Ac-tub
Merge
G
0.45
<1.0E-15
6.0E-10
0.98
Cilia length [µm]
DMSO
ML141
WT
BBS4 KO
BBS4 KO YFP-BBS4
H
0.56
0.0006
0.003
0.37
ARL13B foci frequency
DMSO
ML141
WT
BBS4 KO
BBS4 KO YFP-BBS4
I MEF Bbs4 KO +ML141
B
T
Ac-Tub intensity
ARL13B intensity
Distance [µm]
J
NM_009376.3
sgRNA
PAM
Ift88 WT
Ift88 KO c.248-249insT
K
MEF WT
ST2
GPR161-mCherry DMSO/SAG
WT
Ift88 KO
#1 #2 #3 #4
+IFT88-mNG
IFT88
Actin
130
100
70
40

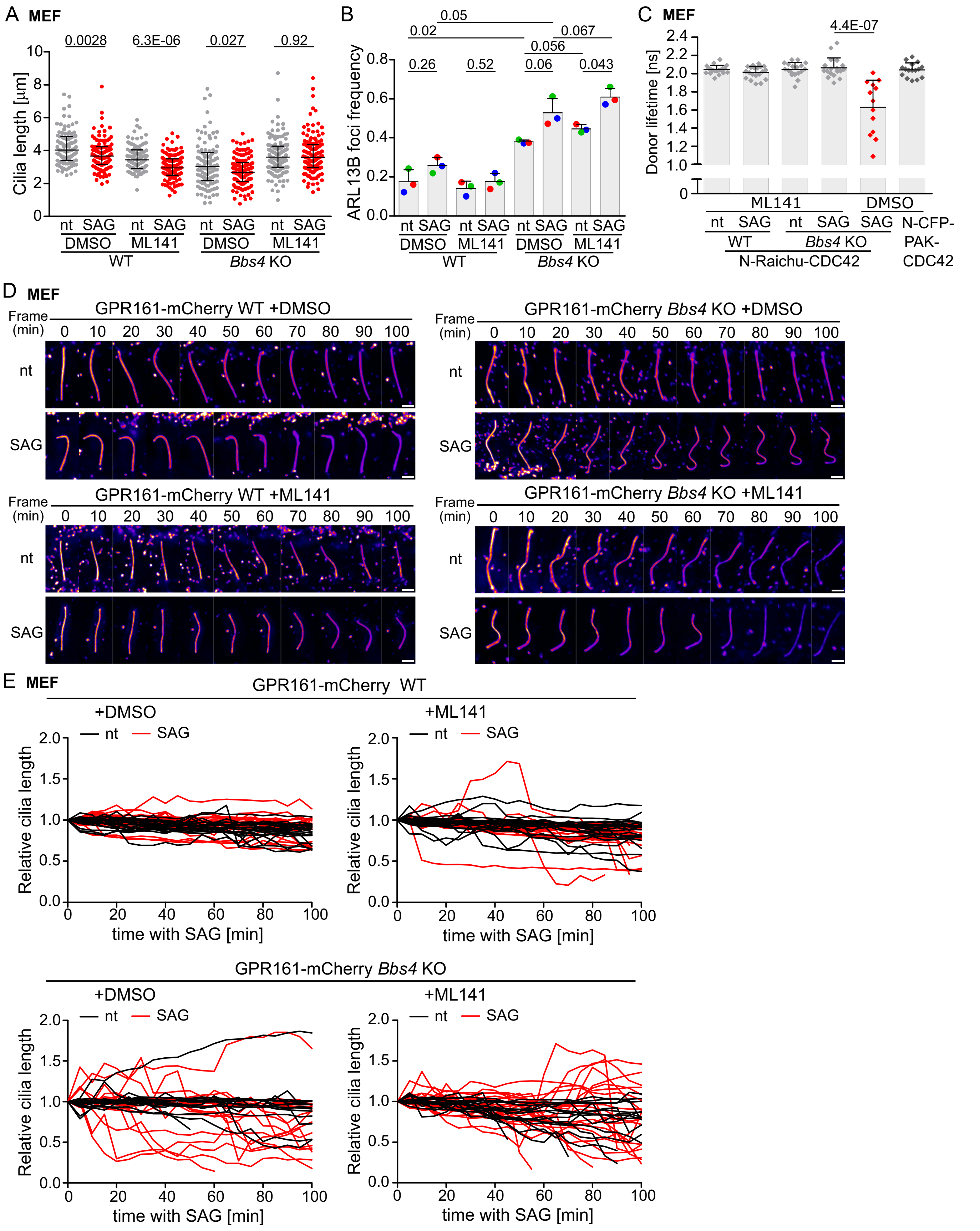
A MEF
0.0028
6.3E-06
0.027
0.92
Cilia length [µm]
nt SAG nt SAG nt SAG nt SAG
DMSO ML141 DMSO ML141
WT
Bbs4 KO
B
ARL13B foci frequency
0.05
0.02
0.067
0.056
0.26
0.52
0.06
0.043
nt SAG nt SAG nt SAG nt SAG
DMSO ML141 DMSO ML141
WT
Bbs4 KO
C MEF
Donor lifetime [ns]
4.4E-07
ML141
DMSO
nt SAG nt SAG SAG
WT
Bbs4 KO
N-Raichu-CDC42
N-CFP-PAK-CDC42
D MEF
GPR161-mCherry WT +DMSO
GPR161-mCherry Bbs4 KO +DMSO
GPR161-mCherry WT +ML141
GPR161-mCherry Bbs4 KO +ML141
Frame (min)
nt
SAG
E MEF
GPR161-mCherry WT
GPR161-mCherry Bbs4 KO
+DMSO
+ML141
nt
SAG
Relative cilia length
time with SAG [min]

 © The Author(s)

Figure EV2. CDC42 hyperactivation drives cilia shortening in BBSome-deficient cells upon SHH triggering.

(**A**) Quantification of the cilia length, in non-treated (nt) and SAG induced—2 h, WT and *Bbs4*$^{KO/KO}$ MEFs concomitantly treated with DMSO or ML141 and stained with antibodies to acetylated tubulin (Ac-tub) and ARL13B. Medians with interquartile range from three independent experiments ($n = 90$–130 cilia). (**B**) Quantification of the frequency of ARL13B foci at the cilia tips in non-treated (nt) and SAG induced—2 h, WT and *Bbs4*$^{KO/KO}$ MEFs concomitantly treated with DMSO or ML141 in (**A**). Mean and SD of three independent experiments ($n = 90$–130 cilia). (**C**) The donor lifetime values extracted from the FRET-FLIM analysis of cilia as a region of interest in non-treated (nt) and SAG induced—2 h, WT and *Bbs4*$^{KO/KO}$ MEFs expressing N-Raichu-CDC42 treated with ML141 or DMSO and values measured for the no-FRET control expressed in WT MEFs treated with DMSO. Mean and SD of three (WT) and four (KO, no-FRET) independent experiments ($n = 13$–20 cilia). (**D**) Representative micrographs of the cilia in non-treated (nt) and SAG induced WT and *Bbs4*$^{KO/KO}$ MEFs expressing GPR161-mCherry, pre-treated for 30 min with DMSO or ML141 and imaged every 1 min for 100 min. Frames were extracted from the live cell videos (17–30 cilia per condition in two independent experiments). Scale bar, 2 μm. (**E**) Plots depict the variable dynamics of the length of the individual cilia in non-treated (nt) and SAG induced WT and *Bbs4*$^{KO/KO}$ MEFs expressing GPR161-mCherry, pre-treated for 30 min with DMSO or ML141 and imaged every 1 min for 100 min in (**D**). The length of the cilium was normalized to the length measured at time 0 min. In total 17–30 cilia were monitored per condition in two independent experiments. Data information: Statistical significance was calculated using the two-tailed Mann–Whitney test (**A**, **C**) and two-tailed paired t-test (**B**) and the obtained *p*-values are indicated.

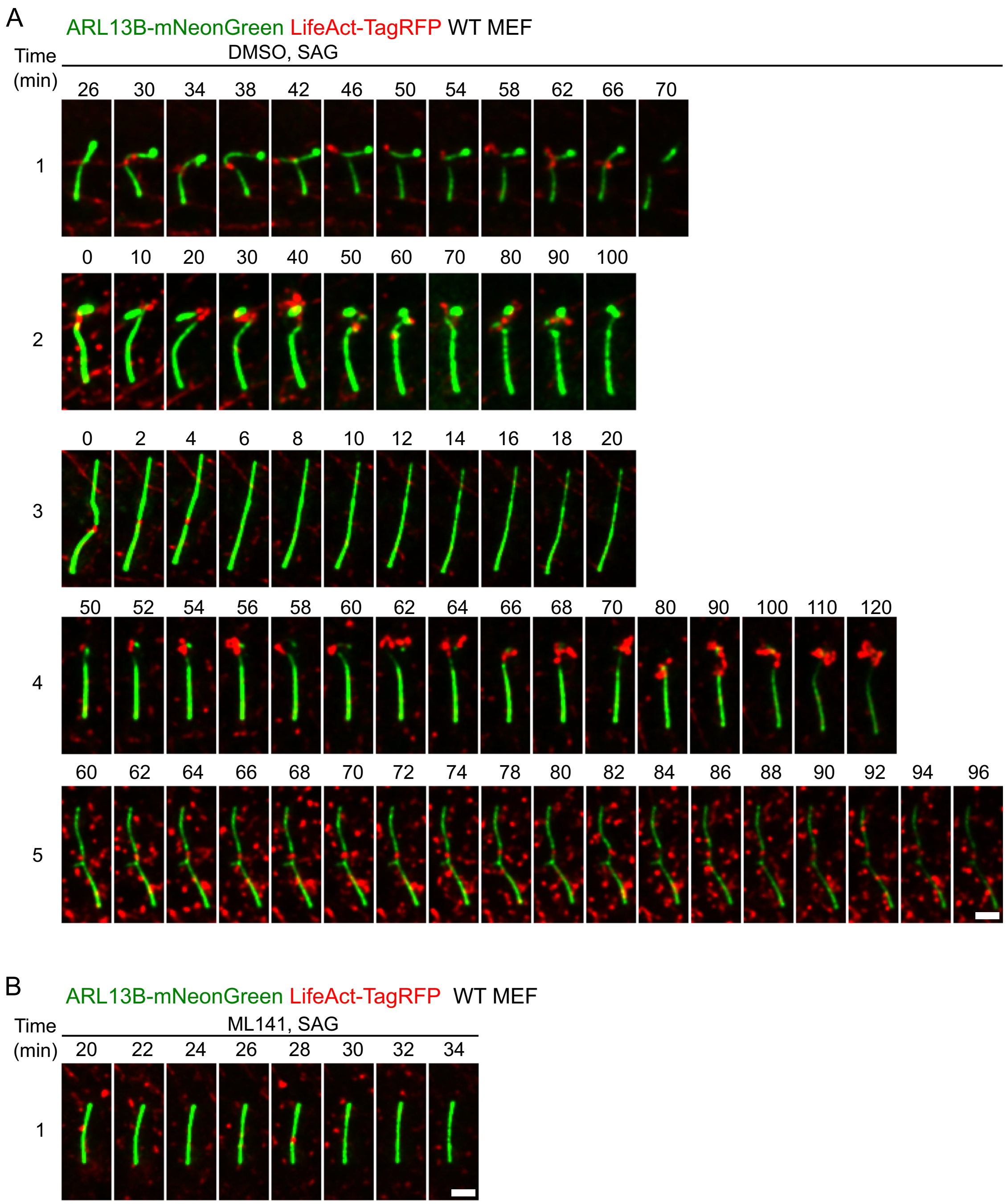

Figure EV3. CDC42 is required for actin polymerization in cilia in WT cells.

(**A**) Representative micrographs show F-actin polymerization events observed in cilia in WT MEFs expressing mNeonGreen-ARL13B (green) and LifeAct-TagRFP (red) treated with SAG and DMSO and imaged for 2 h. Frames were extracted from time-lapse videos (51 in total, 6 actin polymerization events, four independent experiments). Scale bar, 2 µm. (**B**) Representative micrographs show F-actin polymerization events observed in cilia in WT MEFs expressing mNeonGreen-ARL13B (green) and LifeAct-TagRFP (red) treated with SAG and ML141 and imaged for 2 h. Frames were extracted from time-lapse videos (54 in total, 2 actin polymerization events, four independent experiments). Scale bar, 2 µm. Maximum intensity projections of the z-stacks were done using Fiji ImageJ software and the intensities for both channels were adjusted post acquisition for better visualization.

© The Author(s)

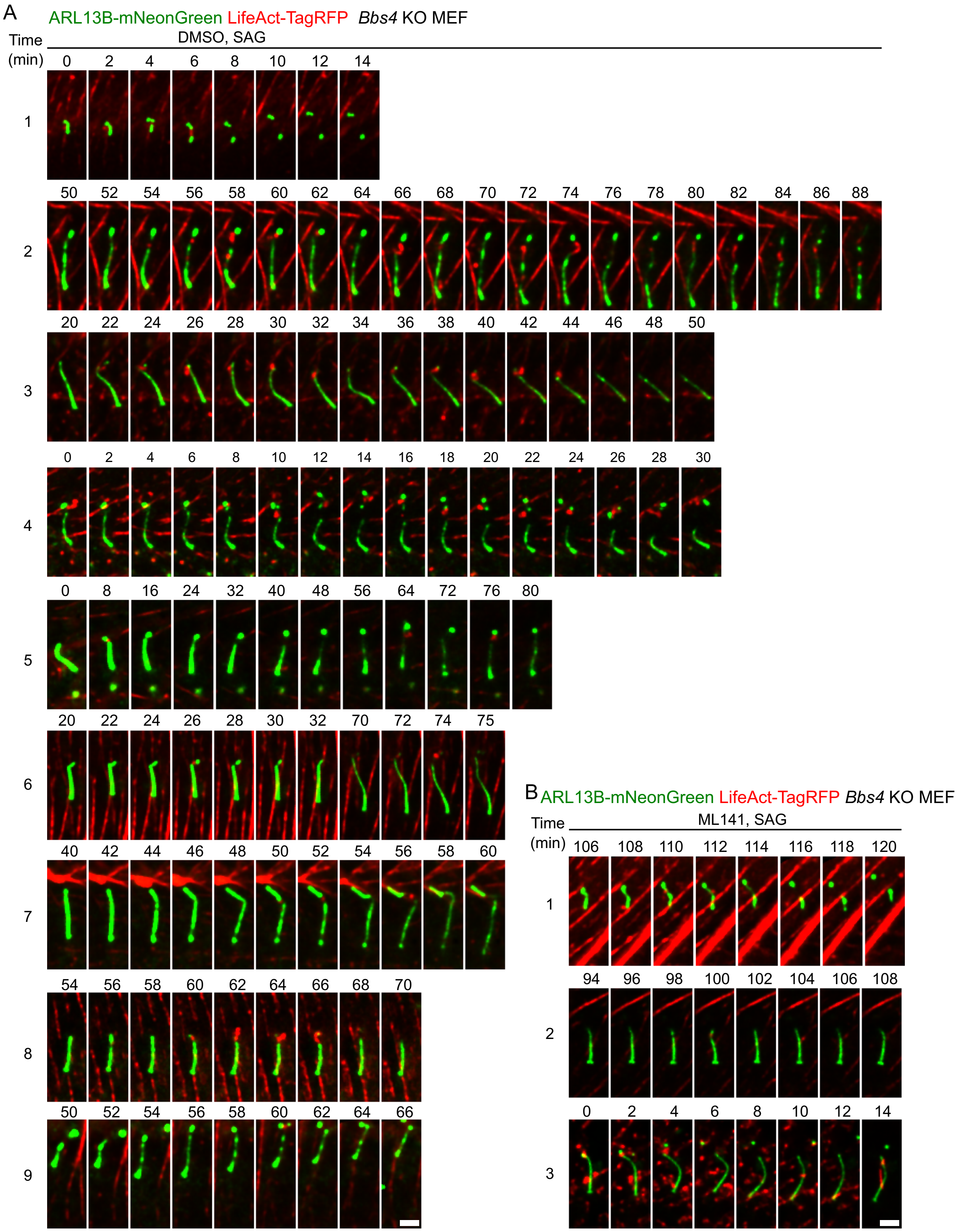
A
ARL13B-mNeonGreen LifeAct-TagRFP Bbs4 KO MEF
Time (min)
DMSO, SAG
0 2 4 6 8 10 12 14
1
50 52 54 56 58 60 62 64 66 68 70 72 74 76 78 80 82 84 86 88
2
20 22 24 26 28 30 32 34 36 38 40 42 44 46 48 50
3
0 2 4 6 8 10 12 14 16 18 20 22 24 26 28 30
4
0 8 16 24 32 40 48 56 64 72 76 80
5
20 22 24 26 28 30 32 70 72 74 75
6
40 42 44 46 48 50 52 54 56 58 60
7
54 56 58 60 62 64 66 68 70
8
50 52 54 56 58 60 62 64 66
9
B
ARL13B-mNeonGreen LifeAct-TagRFP Bbs4 KO MEF
Time (min)
ML141, SAG
106 108 110 112 114 116 118 120
1
94 96 98 100 102 104 106 108
2
0 2 4 6 8 10 12 14
3

Figure EV4. CDC42 is required for actin polymerization in cilia in BBSome-deficient cells.

(**A**) Representative micrographs show F-actin polymerization events observed in cilia in *Bbs4*$^{KO/KO}$ MEFs expressing mNeonGreen-ARL13B (green) and LifeAct-TagRFP (red) treated with SAG and DMSO and imaged for 2 h. Frames were extracted from time-lapse videos (39 in total, 10 actin polymerization events, four independent experiments). Scale bar, 2 μm. (**B**) Representative micrographs show F-actin polymerization events observed in cilia in *Bbs4*$^{KO/KO}$ MEFs expressing mNeonGreen-ARL13B (green) and LifeAct-TagRFP (red) treated with SAG and ML141 and imaged for 2 h. Frames were extracted from time-lapse videos (39 in total, 4 actin polymerization events, four independent experiments). Scale bar, 2 μm. Maximum intensity projections of the z-stacks were done using Fiji ImageJ software and the intensities for both channels were adjusted post acquisition for better visualization.

 © The Author(s)

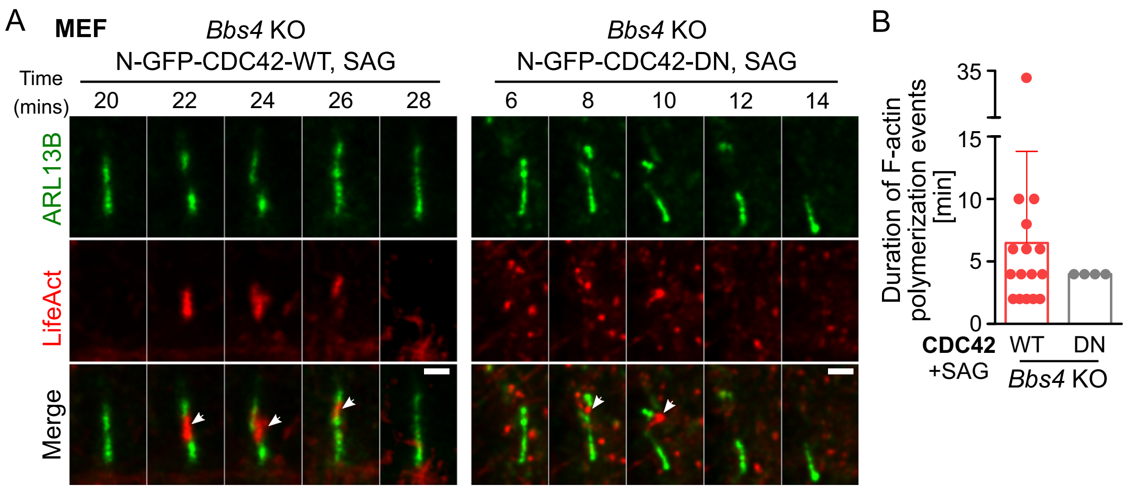

Figure EV5. Activity of cilia localized CDC42 is required for actin polymerization in cilia in BBSome-deficient cells.

(**A**) Representative micrographs depicting ciliary ectocytosis and actin polymerization detected by live cell imaging of *Bbs4*$^{KO/KO}$ MEFs expressing mNG-ARL13B and LifeAct-TagRFP in the presence of WT or DN version of CDC42. The cells were treated with SAG and imaged every 2 min for 2 h. White arrows point to the F-actin polymerization events. Cilia base at bottom. Scale bar, 2 µm. Maximum intensity projections of the z-stacks were done using Fiji ImageJ software and the intensities for both channels were adjusted post acquisition for better visualization. (**B**) The plot depicts the duration of the observed actin polymerization events detected in cilia in the *Bbs4*$^{KO/KO}$ MEFs expressing mNG-ARL13B and LifeAct-TagRFP in the presence of WT or DN version of CDC42 upon treatment with SAG during live cell imaging—six independent experiments in each condition (**A**). Means with SD are shown.

© The Author(s)
